# Host-microbiota interactions and responses of *Metapenaeus ensis* infected with decapod iridescent virus 1

**DOI:** 10.3389/fmicb.2022.1097931

**Published:** 2023-01-13

**Authors:** Minze Liao, Xuzheng Liao, Xinxin Long, Jichen Zhao, Zihao He, Jingyue Zhang, Tingfen Wu, Chengbo Sun

**Affiliations:** ^1^College of Fisheries, Guangdong Ocean University, Zhanjiang, Guangdong, China; ^2^School of Marine Sciences, Sun Yat-sen University, Guangzhou, China; ^3^Institute of Animal Science, Guangdong Academy of Agricultural Sciences, Key Laboratory of Animal Nutrition and Feed Science in South China, Ministry of Agriculture and Rural Affairs, Guangdong Provincial Key Laboratory of Animal Breeding and Nutrition, Guangzhou, China; ^4^Guangdong Provincial Key Laboratory of Pathogenic Biology and Epidemiology for Aquatic Economic Animals, Zhanjiang, Guangdong, China; ^5^Southern Marine Science and Engineering Guangdong Laboratory (Zhanjiang), Zhanjiang, Guangdong, China

**Keywords:** host-microbiota interactions, decapod iridescent virus 1, *Metapenaeus ensis*, intestine microbiota, intestinal immune responses

## Abstract

**Introduction:**

Decapod iridescent virus 1 (DIV1) has caused severe economic losses in shrimp aquaculture. So far, Researchs on DIV1-infected shrimp have mainly focused on the hemocytes immune response, while studies on the host-intestine microbiota interactions during DIV1 infection have been scarce.

**Methods:**

This study determined the lethal concentration 50 (LC_50_) of DIV1 to *Metapenaeus ensis*, preliminarily determining that *M. ensis* could serve as a susceptible object for DIV1. The interactions and responses between the immune and intestine microbiota of shrimp under DIV1 infection were also investigated.

**Results and Discussion:**

DIV1 infection decreases intestine bacterial diversity and alters the composition of intestine microbiota. Specifically, DIV1 infection decreases the abundance of potentially beneficial bacteria (Bacteroidetes, Firmicutes, and Actinobacteria), and significantly increases the abundance of pathogenic bacteria such as *Vibrio* and *Photobacterium*, thereby increasing the risk of secondary bacterial infections. The results of PICRUSt functional prediction showed that altered intestine microbiota induces host metabolism disorders, which could be attributed to the bioenergetic and biosynthetic requirements for DIV1 replication in shrimp. The comparative transcriptomic analysis showed that some metabolic pathways related to host immunity were significantly activated following DIV1 infection, including ncRNA processing and metabolic process, Ascorbate and aldarate metabolism, and Arachidonic acid metabolism. *M. ensis* may against DIV1 infection by enhancing the expression of some immune-related genes, such as Wnt16, heat shock protein 90 (Hsp90) and C-type lectin 3 (Ctl3). Notably, correlation analysis of intestinal microbial variation with host immunity showed that expansion of pathogenic bacteria (*Vibrio* and *Photobacterium*) in DIV1 infection could increased the expression of NF-κB inhibitors cactus-like and Toll interacting protein (Tollip), which may limit the TLR-mediated immune response and ultimately lead to further DIV1 infection.

**Significance and Impact of the Study:**

This study enhances our understanding of the interactions between shrimp immunity and intestinal microbiota. The ultimate goal is to develop novel immune enhancers for shrimp and formulate a safe and effective DIV1 defense strategy.

## 1. Introduction

Intestine microbiota plays an essential role in host health as a coworking collection in the intestine (Ramírez et al., 2018; Negi et al., 2019b; Wu et al., 2021). Previous studies reported that changes in intestine microbiota could impair the host’s defense response to pathogen invasion, thereby affecting the host’s health status (He et al., 2017). It turns out that regulating the composition of the intestinal microbiome through antibiotics, prebiotics, polyphenols, probiotics, or fecal microbiota transplantation can help treat the host (Khan et al., 2021). Thus, a better understanding of host-microbiota interactions and responses to targeted diseases could facilitate the development of novel therapeutic approaches and strategies (Clemente et al., 2012).

Host cells use pattern recognition receptors (PRRs) to recognize two kinds of proteins on microorganism-associated molecular patterns (MAMPs) and pathogen-associated molecular patterns (PAMPs; Dhar and Mohanty, 2020). During pathogenic exposures, host trains PRRs expressing innate cells through intestinal microbial/non-microbial ligands to form a protective mechanism independent of adaptive immunity. Intestine microbiota-derived metabolites and immunomodulatory signals, such as butyrate, acetate, and propionate, tune the immune cells for pro and anti-inflammatory responses, thereby affecting the susceptibility to various diseases (Jia et al., 2018; Negi et al., 2019a). In a related study of grass carp, it was found that intestine microbiota to grass carp reovirus (GCRV)-induced expansion of gram-negative anaerobic *Cetobacterium somerae* can aggravate host inflammatory responses through lipopolysaccharide (LPS)-related NOD-like receptors (NLRs) and toll-like receptors (TLRs) pathways (Xiao et al., 2021). The study of intestine microbiota-immunity associations not only improved our understanding of the interactions between host and intestine microbiota challenged by pathogenic infection but also provided new insights into the ecological defense of disease by controlling the composition of intestine microbiota. However, much less is understood of crustacean immune responses to a dysbiosis of intestine microbiota initiated by virus invasion (Ding et al., 2017). A previous meta-analysis demonstrated that the intestine microbiota of healthy *Litopenaeus vannamei* was distinct from those infected with four diseases, including retardation, mysis mold syndrome, white feces syndrome (WFS), and hepatopancreatic necrosis disease (AHPND; Yu et al., 2018). A previous report showed that White spot syndrome virus (WSSV) affected *L. vannamei* metabolism and immune function by altering their intestinal microbiome composition (Wang J. et al., 2019). Notably, studies have also reported that WSSV infection changes the intestine microbiota of Chinese mitten crabs (Ding et al., 2017). It can be seen that viral diseases have a significant impact on the stability of intestinal microorganisms in crustaceans.

Iridoviruses are a large (~120–200 nm in diameter) icosahedral linear double-stranded DNA viruses that has caused severe mortality and stunted growth in shrimp (Liao et al., 2022). In Xu et al. (2016) discovered a new iridescent virus from *Cherax quadricarinatus* on a farm in Fujian, China, and named *Cherax quadricarinatus* iridovirus (CQIV). In Qiu et al. (2017) isolated shrimp hemocyte iridescent virus (SHIV) from diseased *L. vannamei*, and using intramuscular injection, oral administration and reverse gavage methods to infect *L. vannamei* with SHIV, resulting in a 100% cumulative mortality. In March 2019, the Executive Committee of the International Committee on Taxonomy of Viruses (ICTV) classified SHIV and CQIV as *Decapodiridovirus*, a new genus from the family Iridoviridae. The two viruses are considered to be different strains of the same species, thus proving that the two strains are decapod iridescent virus 1 (DIV1) (Qiu et al., 2019). Recently, DIV1 has posed significant challenges to shrimp farming due to its wide host range and substantial toxicity. Up to now, DIV1 has been detected in a variety of economic shrimp, including *L. vannamei*, *Penaeus monodon*, *Marsupenaeus japonicus* and *Fenneropenaeus merguiensis* (Liao X.Z. et al., 2020; Liao X. et al., 2020; He et al., 2021a,b).

Recently, we discovered a new susceptible shrimp with DIV1——*Metapenaeus ensis*. *M. ensis* is one of the most important aquaculture shrimps in China (Cui et al., 2013). At the same time, viral infections have restricted the continued growth of *M. ensis* aquaculture, posing significant challenges to the shrimp industry (Liao et al., 2022). The study of intestine microbiota could provide effective theoretical guidance for prevention and control of DIV1 infection. After DIV1 infection, most decapod crustaceans, such as *Macrobrachium rosenbergii*, *L. vannamei*, *Exopalaemon carinicauda* and *P. monodon*, have clinical signs of empty stomach and intestine (Qiu et al., 2017; Chen et al., 2019; Qiu et al., 2019; He et al., 2021a). It is speculated that the composition of intestine microbes in decapod crustaceans will also change after DIV1 infection. Several studies have reported that DIV1 infection caused visible damage to the shrimp intestine, resulting in intestine immune system disorder and microbiota function changes (Duan et al., 2018; He et al., 2022). Therefore, RNA-seq was applied in this study to elucidate changes in the major pathways in the intestine of *M. ensis* under DIV1 infection, based on lethal concentration 50 (LC_50_) test results. Meanwhile, intestinal microbial community changes were examined using 16S rRNA sequencing. This new information contributes to a better understanding of the response of the intestinal immune function of *M. ensis* to DIV1 infection, aiming to provide a theoretical basis for the further development of new shrimp immune enhancers and microbial preparations.

## 2. Materials and methods

### 2.1. Shrimp culture

The study protocol was approved by the Ethics Review Board of the Institutional Animal Care and Use Committee at Guangdong Ocean University. Healthy *M. ensis* were purchased from a local shrimp farm in Zhanjiang City, Guangdong Province, China. *M. ensis* (body weight 10.9 ± 2.4 g) adapted for a week in 300 l fiberglass drums with seawater at salinity of 30.62 ± 0.69 ‰, pH of 7.96 ± 0.05, temperature of 27.61 ± 0.69°C. Before the LC_50_ test, *M. ensis* were randomly selected for PCR amplification to confirm WSSV, infectious hypodermal and hematopoietic necrosis virus (IHHNV) and DIV1 were not present. These viral assays were performed using the methods in a previously published paper (Tang et al., 2007; Qiu et al., 2017; Siddique et al., 2018). The primers used for virus detection are shown in Supplementary Table S1.

### 2.2. LC_50_ test and sample collection

DIV1 was obtained from DIV1 infected tissues preserved in our laboratory. These tissues were from the same source as previously reported (Liao X. et al., 2020). Preparation of DIV1 inoculum was performed using the method in previous studies (Chen et al., 2019). The viral loads of the samples were detected by real-time PCR in a CFX Connect™ Real-Time system (Bio-Rad, United States) using the primers qRT-DIV1-F, qRT-DIV1-R, and TaqMan Probe (Supplementary Table S1) with the following procedure: denaturation at 95°C for 30 s, followed by 40 cycles at 95°C for 5 s and 60°C for 30 s (Sun et al., 2013). In the LC_50_ assay, *M. ensis* were randomly divided into seven groups of three replicates (n = 30). In six of these groups, shrimps were injected intramuscularly with 50 μl of viral inoculum at the third abdominal segment with concentrations of 5.85 × 10^9^, 5.85 × 10^8^, 5.85 × 10^7^, 5.85 × 10^6^, 5.85 × 10^5^ and 5.85 × 10^4^ copies/μg DNA. 50 μl of PBS buffer (pH 7.4) was injected in the PBS group. After injection, shrimps were placed into a 300 L fiberglass drum for observation. The cumulative survival rate of *M. ensis* was recorded every 4 h. The dead shrimp were removed to avoid secondary infection. The LC_50_ was analyzed by probit calculation using the Bliss method (Bliss, 1939).

**Figure 1:**
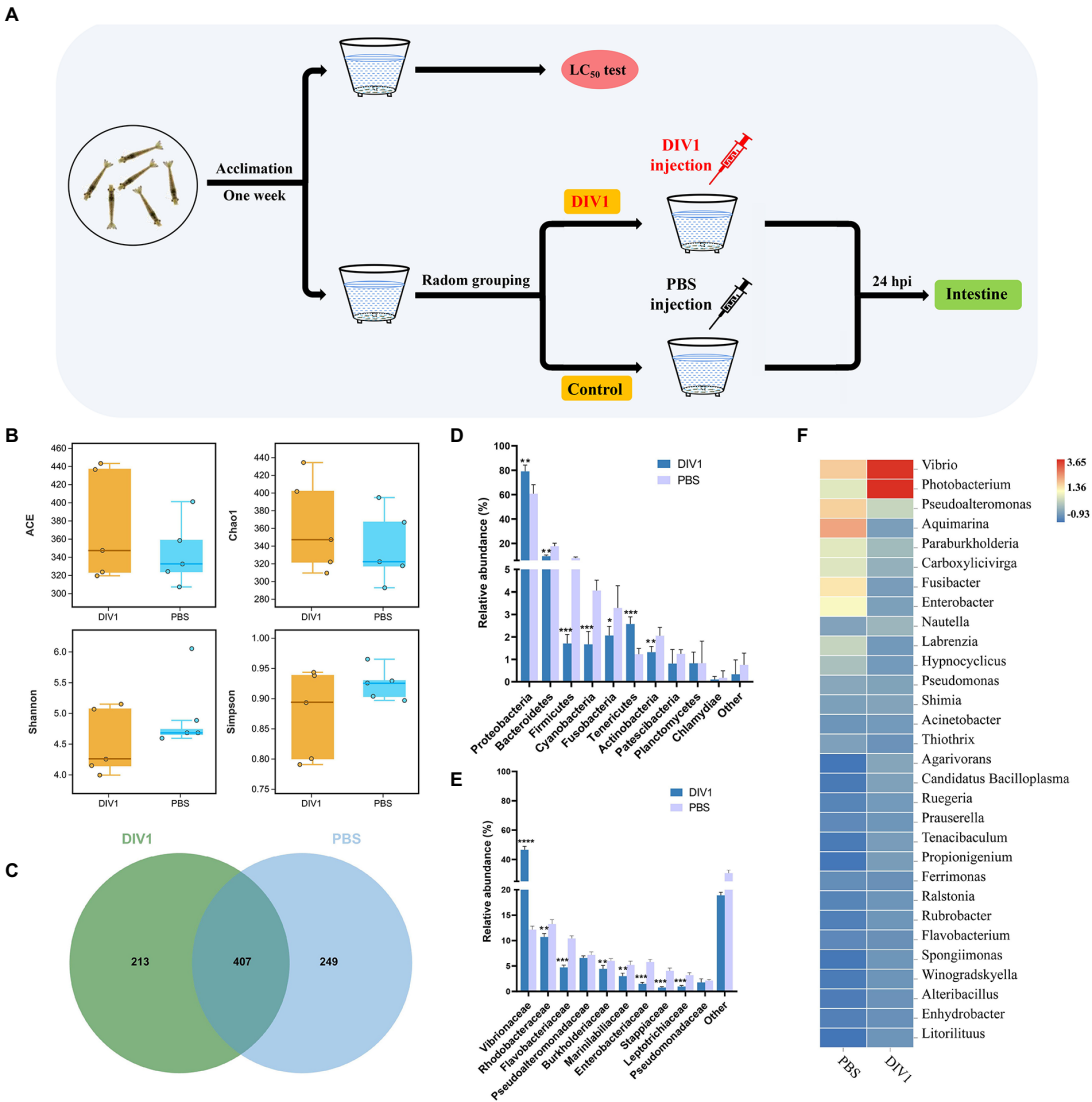
The diversity and bacterial composition of intestinal microbiota in *M. ensis* after DIV1 infection. **(A)** Schematic diagram of the experimental setup. **(B)** Alpha diversity indices for the DIV1-infected and PBS groups. Bars indicate mean ± S.D. (*n* = 5). **(C)** Venn figure showing the unique and shared OTUs of microbiota in DIV1-infected and PBS groups. Display the relative abundance of intestine microbial at the phylum **(D)** and family **(E)** classification level. Bars are shown as mean ± S.D. (*n* = 5). * indicate significant differences between groups. *p < 0.05, **p < 0.01, ***p < 0.001 and ****p < 0.0001. **(F)** Heatmap analysis of the top 30 microorganisms at the genus level. Blue colors indicate lower abundance, and red colors indicate higher abundance. Standardized by column.

*M. ensis* for the challenge experiment was randomly divided into DIV1-infected group and PBS group. The weight of *M. ensis* and culture conditions were the same as in the LC_50_ test. According to the results of LC_50_ test, the LC_50_ of DIV1 infection in *M. ensis* is 5.85 × 10^9^ copies/μg DNA at 24 hpi. Thus, DIV1-infected group was injected intramuscularly with 50 μl of DIV1 inoculum of 5.85 × 10^9^ copies/μg DNA. The PBS group was injected with 50 μl of PBS buffer. Shrimp intestinal samples were collected under aseptic conditions at 24 h post injection (hpi) according to the previously described method (Rungrassamee et al., 2016; Figure 1A). The intestines of three individuals in the same group were pooled into one sample, each group containing five duplicate microbial samples and three duplicate transcriptome samples.

### 2.3. Intestine microbiota analysis

Microbial DNA was extracted by the DNeasy PowerSoil Kit (QIAGEN) and its quantity and quality were measured by Nanodrop spectrophotometer. The V3-V4 fragment of the 16S rRNA gene was amplified using primer pairs 341F (5′-CCTACGGGNGGCWGCAG-3′) and 806R (5′-GGACTACHVGGGTWTCTAAT-3′; Guo et al., 2017). Afterwards, the PCR fragments were evaluated using 2% agarose gels. After PCR purification, the generated sequencing libraries were sequenced on Illumina NovaSeq 6000. Raw reads for all samples has been uploaded to the NCBI Sequence Read Archive database with the accession number SRP393433.

The raw tags were analyzed and filtered to obtain high quality clean tags (Bokulich et al., 2013). The UCHIME algorithm was used to detect and remove all chimeric sequences from the clean tags, and finally effective tags were obtained for further analysis (Edgar et al., 2011). Clustering operational taxonomic units (OTUs) by Uparse (version 9.2.64) and performing species annotation and abundance analysis. Stacked bar charts of microbial community abundance were generated using the ggplot2 package of R project (version 2.2.1). Heat maps of genus abundance were constructed using the pheatmap package (version 1.0.12) from the R project. Alpha diversity indices (Good’s coverage, Shannon, Simpson, ACE and Chao 1) and beta diversity indices were calculated using quantitative insights into microbiota ecology (QIIME; version 1.9.1). PCoA and Venn diagrams were generated using R software (version 2.15.3). Exploring the differences in community structure between two groups of samples using LEfSe method. Differential functional information of KEGG pathways (level 3) was predicted using PICRUSt software (version 2.1.4).

### 2.4. Intestine transcriptome analysis

#### 2.4.1. RNA extraction, library construction, and sequencing

Total RNA was extracted from DIV1-infected and PBS groups by Trizol method. rRNA was removed by Ribo-ZeroTM Magnetic Kit (Epicentre, United States) and mRNA was enriched by Oligo (dT) magnetic beads. First cDNA strand was synthesized in the M-MuLV reverse transcriptase system using mRNA as template, followed by the second cDNA strand was synthesized in the DNA polymerase I system using dNTPs as raw material. The double-stranded cDNA was purified, ligated with a sequence adapter and screened for approximately 200 bp of cDNA for PCR amplification, and the PCR product was purified again with 10Ampre XP beads to obtain a library. Finally, the constructed libraries were sequenced on the Illumina HiSeq2500 platform.

#### 2.4.2. Transcriptome assembly and functional annotation

To obtain clean reads, raw reads from sequencing were filtered to remove low quality reads, connector contamination and ambiguous reads (‘N’ content >10%). Subsequently, samples from the DIV1-infected and PBS groups were *de novo* assembly using Trinity software (Grabherr et al., 2011). The integrity of the assembly was evaluated by BUSCO (version 3.0.2) and BUSCO arthropod dataset (Simão et al., 2015). The unigene sequences were aligned with five available databases at NCBI using the BLASTx program with an E-value threshold of 1e-5, including Nr^1^, Swiss-Prot^2^, GO^3^, KOG^4^ and KEGG^5^.

#### 2.4.3. Identification of differentially expressed genes (DEGs)

The FPKM (Fragment per kilobase of transcript per million mapped reads) values was used to quantify genes expression abundance and variation. After obtaining FPKM values for all genes, the DESeq2 software (Love et al., 2014) was used to analyze differentially expressed RNAs from two groups. This study uses false discovery rate (FDR) as a key indicator for screening differentially expressed genes (DEGs). Genes with the parameter of FDR < 0.05 and |log2 (fold change)| ≥ 2 were considered as DEGs. Moreover, further analysis of subsequent functional enrichment of DEGs using GO and KEGG databases.

#### 2.4.4. Validation of RNA-seq profiles by qPCR

Twelve DEGs (six up regulated genes and six down regulated genes) in *M. ensis* intestinal transcriptome were selected to verify the Illumina sequencing results. Primers were designed using Primer 5, which information is listed in Supplementary Table S1. Before the Real-time PCR experiment, cDNA was generated by reverse transcription of template RNA using 5X All-in-One RT Master Mix (Applied Biological Materials, Vancouver, BC, Canada). RT-qPCR was subsequently carried out using the SYBR® Premix EX Taq™ II (Tli RNase H Plus - Takara Bio, Japan) kit. All selected DEGs were verified by RT-qPCR using the CFX Connect™ Real-Time system (Bio-Rad, United States). The EF1α of *M. ensis* served as an internal control and normalized the expression level of each gene. The relative expression ratio of the target gene versus EF1α was calculated by the 2^−△△CT^ method (Livak and Schmittgen, 2001).

### 2.5. Correlation analysis of intestinal microbial and immune-related DEGs

Three transcriptome samples and three microbial samples were paired in correlation analysis. Among them, the paired samples from transcriptome and microbiome were obtained from the same samples of shrimp. Pearson correlation analysis of intestine dominant bacteria with immune-related DEGs was performed using R (version 3.5.1). The correlation heatmap was generated using the pheatmap package in R. The correlation coefficient and value of *p* threshold were not set. *p* < 0.05 was considered statistically significant, *p* < 0.01 was very significant, and *p* < 0.001 was extremely significant.

### 2.6. Statistical analysis

The data are expressed as mean ± standard deviation (SD). One-way analysis of variance (ANOVA) and Duncan multiple range tests were used for statistical analysis and evaluate whether there were significant differences between these data (*p* < 0.05). Permutational multivariate analysis of variance (PERMANOVA) was used to evaluate the significance of community differences based on Bray-Curtis distance.

## 3. Results

### 3.1. LC_50_ of DIV1 for *Metapenaeus ensis*

**Figure 2:**
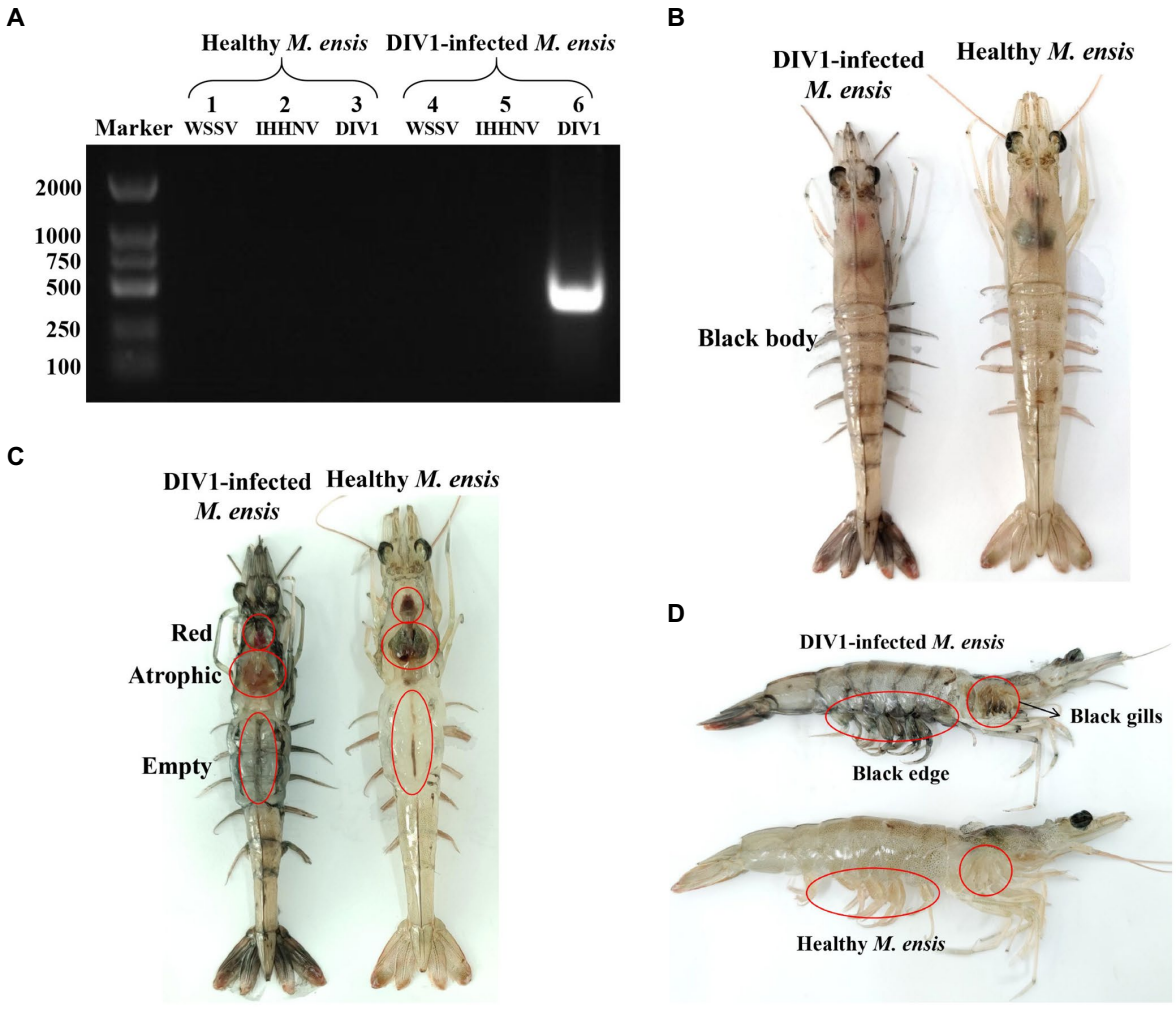
Clinical symptoms and virus detection of *M. ensis.* **(A)** Virus detection of healthy and infected *M. ensis* used for LC_50_ test. Marker: DL2000 molecular mass marker; Lane 1–3: PCR amplified products used for WSSV, IHHNV, and DIV1 detection in healthy *M*. *ensis*; lane 4–6: PCR amplified products used for WSSV, IHHNV, and DIV1 detection in DIV1-infected *M*. *ensis*. **(B–D)** Clinical symptoms of DIV1-infected *M. ensis.*

**Figure 3:**
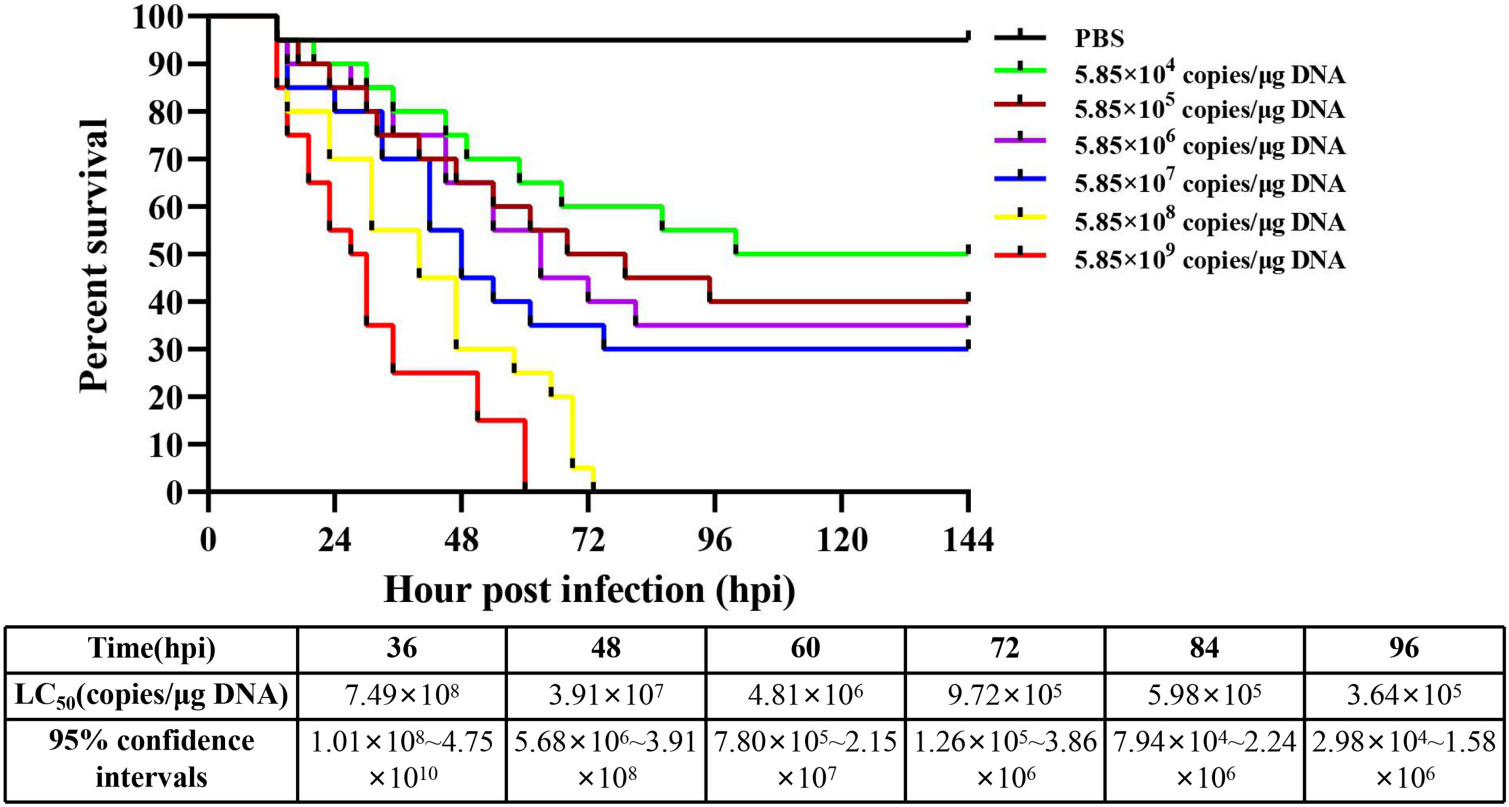
Cumulative survival of *M. ensis* after DIV1 injection and LC_50_ test. Seven groups of healthy *M. ensis* were intramuscularly injected with 50 μl of DIV1 inoculum at six concentrations and PBS as a control.

The detection results showed that the three known common shrimp pathogens, including WSSV, IHHNV, and DIV1, were all negative in cultured shrimp samples. Only DIV1 was found in the infected *M. ensis* used for the DIV1 inoculation (Figure 2A). Compared with healthy *M. ensis*, DIV1-infected *M. ensis* had apparent disease symptoms, including black body, soft shell, red stomach, empty intestine and atrophy of the hepatopancreas with yellowing (Figures 2B,C). In addition, part of the dead *M. ensis* presented symptoms of black gills and black edges of the abdominal shell (Figure 2D). As shown in Figure 3, the survival rates of *M. ensis* were assessed after exposure to different doses of DIV1. The DIV1-induced shrimp mortality rate increased with the increasing concentrations of inoculated virus. *M. ensis* injected with 5.85 × 10^8^ and 5.85 × 10^9^ copies/μg DNA DIV1 supernatant had a mortality rate of 100% at 73 hpi and 60 hpi, respectively, and the mortality rate of other injection doses remained stable at 110 hpi. Probit analysis indicated that the LC_50_ values for DIV1 determined are 7.49 × 10^8^, 3.91 × 10^7^, 4.81 × 10^6^, 9.72 × 10^5^, 9.72 × 10^5^, and 3.64 × 10^5^ copies/μg DNA for 36, 48, 60, 72, 84 and 96 h after injection, respectively.

### 3.2. Intestine microbiota analysis

#### 3.2.1. Richness and diversity

A total of 1,295,380 raw reads were obtained from intestine microbiota of *M. ensis* by 16S rRNA Illumina sequencing, with an average of 128,981 clean reads per sample after quality control and read assembly; the amount of sequencing data was sufficient (Supplementary Table S2). Although there was no significant differences among the DIV1-infected and PBS groups (*p* = 0.06 ~ 0.38 > 0.05), community richness indices (ACE and Chao1) were increased in DIV1-infected group. In contrast, the community diversity indices (Simpson and Shannon) decreased (Figure 1B). Venn figure demonstrates that PBS group contained 249 core OTUs, while DIV1-infected group had 213 core OTUs. A total of 407 OUTs were shared between two groups (Figure 1C), accounting for 31.90% of identified OTUs.

#### 3.2.2. Changes in the intestine bacterial composition

The 16S rRNA genes in the intestine microbiota of *M. ensis* were sequenced to study bacterial community variations induced by DIV1 infection. At the phylum classification level, the DIV1-infected and PBS groups were mainly composed of Proteobacteria, Bacteroidetes, Firmicutes, Cyanobacteria, Tenericutes, and Actinobacteria (Figure 1D). Of those, the abundances of Proteobacteria (79.11%) and Tenericutes (2.58%) increased significantly after DIV1 infection (*p* < 0.01). In contrast, the abundances of Bacteroidetes (9.48%), Firmicutes (1.71%), Cyanobacteria (1.67%), and Actinobacteria (1.32%) significantly decreased following DIV1 infection (*p* < 0.01). At the top ten families classification level, excepting the abundance of Vibrionaceae (46.58%) in the DIV1 infection group, which is significantly higher than the PBS group (12.15%), the abundance of other microorganisms is lower than the PBS group (Figure 1E). Notably, the relative abundance of Vibrionaceae in the intestinal of *M. ensis* significantly increased after infection with DIV1 (*p* < 0.01), almost four times as high as in the PBS group. Distinctions in the composition of bacterial communities were also observed at the genus level, with the abundances of *Vibrio* (23.18%) and *Photobacterium* (23.40%) more dominant in the DIV1-infected group, whereas the abundances of *Vibrio* and *Photobacterium* in PBS group was only 7.25 and 4.89% (Figure 1F). Supplementary Table S3 shows detail informations of relative abundance and *p* value of the top 10 dominant bacterial in phylum, family, and genus between two groups.

**Figure 4:**
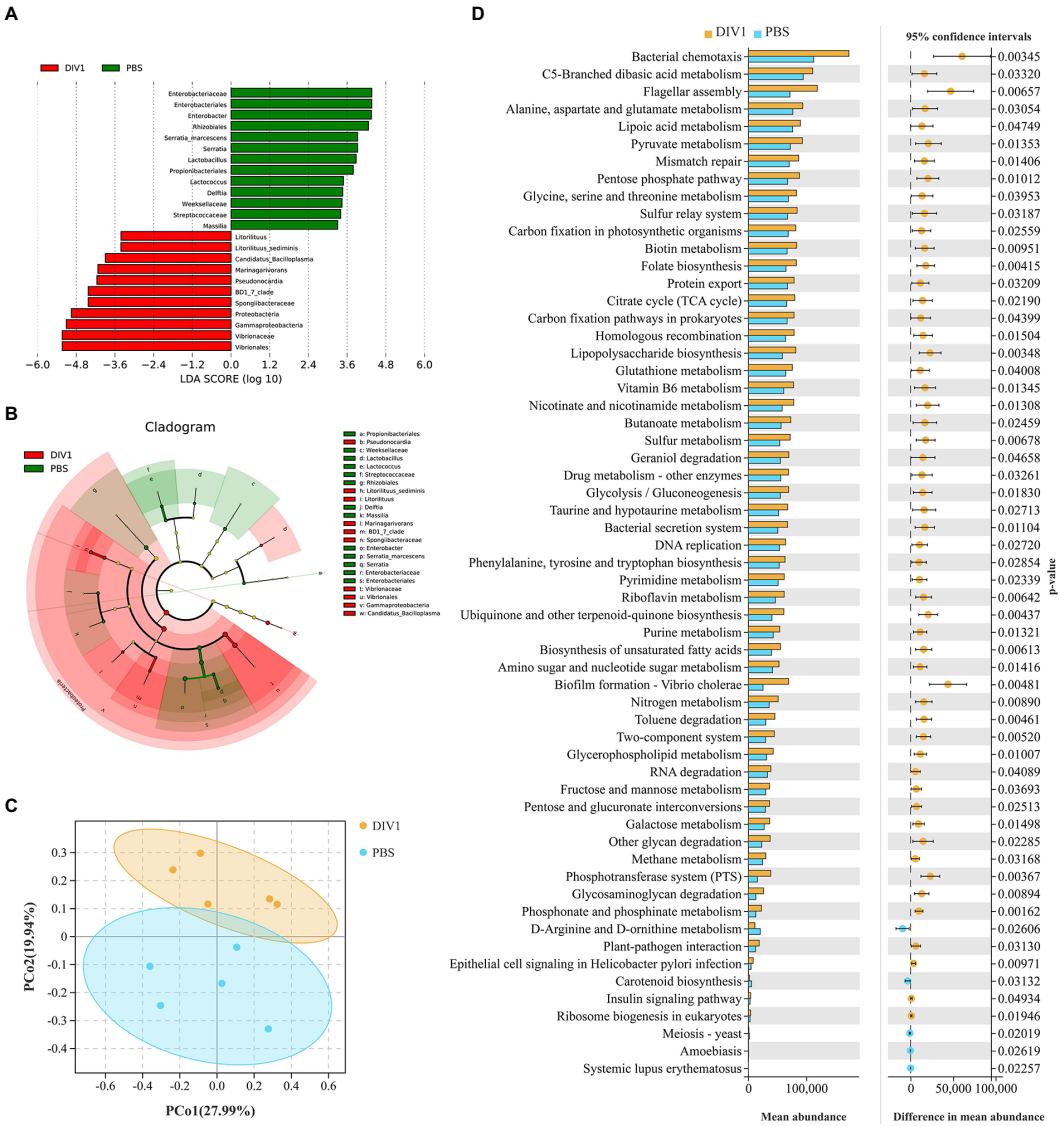
Intergroup differences and PCoA in the relative abundance of intestinal microbial communities between DIV1-infected and PBS groups. **(A)** LDA score of LEfSe. Only taxa with LDA value (influence value of linear discriminant analysis) higher than two were shown. **(B)** Lefse cladogram. Evolutionary branch graph of differential bacterial communities or species. Green: bacterial taxa enriched in PBS group; red: bacterial taxa enriched in DIV1 group; yellow: no significant differences. **(C)** PCoA plot shows the microbial diversity of samples. Samples from the same group were clustered closer. **(D)** Differential functional information between DIV1-infected and PBS groups (KEGG level 3). The left half of the figure: the y-axis represents the differential function, and x-axis represents the abundance of the differential function. The right half of the figure: the x-axis shows the confidence interval range of functional abundance difference between groups, colour indicates the grouping of high abundance, and the y-axis is the *p* value.

LEfSe (Linear discriminant analysis Effect Size) analysis was used to screen microbes differentially among species. The bar chart indicates that 24 specific taxa were identified, with 11 taxa in the DIV1 infection group and 13 taxa in the PBS group (Figure 4A). Notably, Vibrionales and Vibrionaceae were significantly increased following *M. ensis* infection with DIV1. Evolutionary branch diagrams of LEfSe analysis based on classification information indicated 23 differential bacterial taxa that could distinguish two groups. Among them, Vibrionales, Vibrionaceae, and Gammaproteobacteria are included in these 23 differential bacterial taxa (Figure 4B). Furthermore, the PERMANOVA and PCoA analysis based on Bray-Curtis showed that the DIV1-infected samples were separated from the PBS samples (Figure 4C), indicating that the community composition of intestinal microflora was significantly different between two groups (*p* < 0.05) (Supplementary Table S4).

#### 3.2.3. Functional analysis of the intestinal microbiota

PICRUSt is a bioinformatics software for metagenomic functional prediction based on marker gene (e.g., 16S rDNA). In this study, PICRUSt software was used to predict the metagenomic potential of the intestinal environment between DIV1 infection and PBS groups based on KEGG database according to 16S rRNA sequencing data (Langille et al., 2013). Figure 4D shows the apparent changes in KEGG level 3 in the differential function. The results showed that the mean abundance of five metabolism-related pathways is significantly increased following DIV1 infection, including Alanine, aspartate and glutamate metabolism, Lipoic acid metabolism, Pyruvate metabolism, Nicotinate and nicotinamide metabolism, and Pyrimidine metabolism (*p* < 0.05). Furthermore, the mean abundance of Flagellar assembly and Biofilm formation - Vibrio cholerae pathways related to bacterial pathogenicity was extremely significantly increased after DIV1 infection (*p* < 0.01), while Carotenoid biosynthesis was significantly decreased (*p* < 0.05).

### 3.3. Intestine transcriptome analysis

#### 3.3.1. Transcriptome sequencing, *de novo* assembly and annotation

To determine the transcriptome profile of *M. ensis* intestine under DIV1 infection, the Illumina RNA-seq was applied for *M. ensis* intestine samples from DIV1-infected and PBS infected groups. After quality filtering, the DIV1-infected and PBS groups yielded 93,073,404 and 43,194,564 clean reads, respectively. PBS groups got 6.44 Gb nucleotides, whereas the DIV1-infected groups obtained 13.90 Gb nucleotides. The average guanine-cytosine (GC) content of clean reads was 45.87% in DIV1-infected group and 47.52% in PBS group. Detailed information on sequencing and assembly is provided in Supplementary Table S5. All sequencing reads are submitted to the NCBI Sequence Read Archive (SRA^6^) and can be found under accession number SRP394201. After removing the redundancies and aligning the assembled contigs, 42,827 unigenes were obtained (N50 Length = 2,377 bp). Supplementary Figure S1A shows the length and size distribution of unigeness in PBS and DIV1-infected groups. Among these unigenes, the majority are 200–300 nt (11,457, 26.75%) in length, followed by 300–400 nt (5,834, 13.62%) and 4,083 unigenes (9.53%) ≥3,000 nt. To further test the integrity of transcriptome, *M. ensis* intestinal transcriptome was compared with 978 conserved arthropod genes by Benchmarking Universal Single-Copy Orthologs (BUSCO). The results showed that 958 genes in *M. ensis* intestinal transcriptome encoded complete proteins. Among these genes, 854 genes were complete and single-copy BUSCOs, 104 genes were complete and duplicated BUSCOs, 9 genes were fragmented BUSCOs prototypes, and 11 genes were missing BUSCOs altogether (Supplementary Figure S1B). To gain comprehensive functional information, the unigenes obtained from RNA-seq were annotated in five major functional databases (Supplementary Figure S1C). These databases included Nr (21,126 unigenes), SwissProt (15,163 unigenes), KOG (13,216 unigenes), KEGG (20,193 unigenes), and GO (11,496 unigenes). The analyses revealed that Nr had the largest number of homologous sequences to assembled unigenes among the 81,194 unigenes.

Nr annotation revealed that over 74.64% of the total unigenes matched with the sequences of ten top-hit species, including *L. vannamei* (60.68%), *Homo sapiens* (7.58%), *Pan troglodytes* (1.36%), *Hyalella azteca* (0.89%), *Trinorchestia longiramus* (0.75%), *Pongo abelii* (0.73%), *Armadillidium nasatum* (0.69%), *Paragonimus westermani* (0.68%), *Mus musculus* (0.67%), and *M. japonicus* (0.61%) (Supplementary Figure S2A). Through GO annotation, 11,496 unigenes were enriched in 68 GO terms (level 2), divided into three overarching categories: cellular components (23 subcategories), molecular functions (18 subcategories), and biological process (27 subcategories) (Supplementary Figure S2B). In the category of “biological processes,” the largest number of unigenes participated in “cellular process” and “single-organism process.” The majority of unigenes in “cellular component” category were involved in “cell” and “cell part.” As for the “molecular functions” category, “binding” and “catalytic activity” were the dominant groups. The public KOG database was then used to explore the orthologous functions of the unigenes further. In this study, 13,216 unigenes were successfully annotated into the KOG database, distributed in 25 categories (Supplementary Figure S2C). Among the functional classification categories, “General function prediction only” (15.99%) was the largest group, “Signal transduction mechanisms” (12.00%) and “Posttranslational modification, protein turnover, chaperones” (10.05%) being the next largest groups. To determine the biological processes of unigenes, 20,193 unigenes were annotated with the KEGG database and mapped to six major groups in KEGG level 1, including organismal systems, metabolism, human diseases, genetic information processing, environmental information processing, and cellular processes (Supplementary Figure S3). These annotated unigenes were further divided into 45 subcategories. The largest subcategory group was infectious diseases (4,184 unigenes), followed by signal transduction (3,634 unigenes) and cancers (2,953 unigenes).

#### 3.3.2. Functional characterization and identification of DEGs

**Figure 5:**
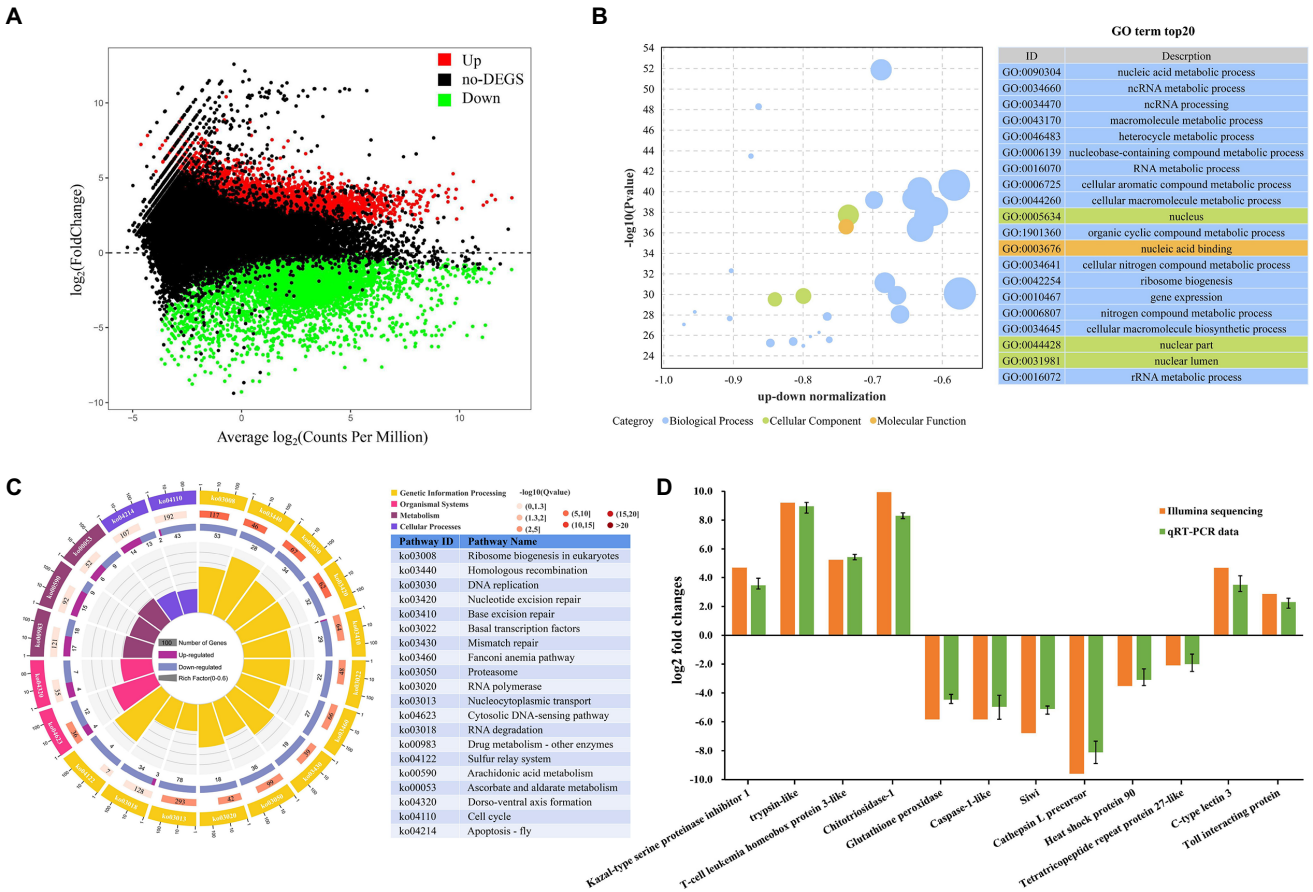
*Metapenaeus ensis* intestinal transcriptomic responses after DIV1 infection. **(A)** MA plots between DEGs in DIV1-infected and PBS group. The x-axis represents the average expression level. The y-axis represents the logarithm of multiple gene expression differences between two samples. The green and red dots represent genes with significant differences in expression. The Green dots represent down regulated gene expression, red dots represent up regulated gene expression, and black dots represent genes with no significant differences in expression. **(B)** Top 20 significantly enriched GO terms. Different colors show different GO categories. The x-axis represents the up-down normalization, and the y-axis represents −log10 (*p value*). **(C)** The results of DEGs in KEGG pathway enrichment analysis. Four laps from outside to inside, the first lap indicates the top 20 KEGG terms, and different colors indicates different classifications, with the number of genes corresponding to the outer lap. The second lap represents the number of genes in the genome background and the q-value for DEGs enrichment in specific biological processes. The more genes, the longer the bars. The third lap indicates the total number of DEGs, including up regulated genes (deep purple) and down regulated genes (light purple). The fourth lap represents the enrichment factor of each KEGG term. **(D)** Comparison of RNA-Seq and RT-qPCR expression data of 12 selected genes.

In this study, FDR <0.05 was set as the cutoff value and |log2 (FC)| ≥2 was used as the threshold to select DEGs among *M. ensis* intestinal unigenes in DIV1-infected and PBS groups. The results showed that a total of 5,956 DEGs were identified, including 2,466 up regulated genes and 3,490 down regulated genes (Figure 5A). The DEGs were found to have many biological functions by the Nr database annotation. Some genes are implicated in innate immune defense, such as Dual oxidase, Wnt16, C-type lectin 3 (Ctl3), Toll interacting protein (Tollip), caspase 2, caspase 4, heat shock protein 90 (Hsp90), cathepsin B, NF-κB inhibitor cactus-like, and Endoplasmin (Supplementary Table S6).

All the detected DEGs were annotated through the GO and KEGG databases to evaluate the biological function of DEGs further. In the GO enrichment analysis, a total of 3,923 DEGs expressed in the DIV1-infected group were divided into three main functional categories with 64 subcategories, that is, biological progress (26 subcategories), cellular component (23 subcategories), and molecular function (15 subcategories). The top 20 GO terms, selected by *p* value, were shown in Figure 5B. Most of the corresponding DEGs were enriched in the nucleus and nuclear region in the cellular component category. In the molecular function category, the corresponding DEGs were mainly enriched in nucleic acid binding and heterocyclic compound binding. Notably, the nucleic acid metabolic process (1,586 DEGs), ncRNA metabolic process (439 DEGs), and ncRNA processing (350 DEGs) are the three most enrichment subclasses in the biological process.

The KEGG pathway enrichment analyses further investigated the biological effects of DEGs. In this study, 1,537 DEGs were enriched into 339 pathways. The top 20 significantly different pathways influenced by DIV1 infection were shown in Figure 5C. Some pathways that may be associated with the immunity of *M. ensis* were also identified through KEGG enrichment, such as Homologous recombination (twenty-eight down regulated genes), Base excision repair (one up regulated gene and twenty-nine down regulated genes), Mismatch repair (nineteen down regulated genes), Proteasome (thirty-six down regulated genes), RNA polymerase (eighteen down regulated genes), Nucleocytoplasmic transport (seventy-eight down regulated genes), Cytosolic DNA-sensing pathway (four up regulated and twelve down regulated genes), Cell cycle (two up regulated and forty-three down regulated genes), Apoptosis-fly (fourteen up regulated and thirteen down regulated genes), Arachidonic acid metabolism (fifteen up regulated and nine down regulated genes), and Ascorbate and aldarate metabolism (six up regulated and nine down regulated genes).

#### 3.3.3. Validation of RNA-seq results by qRT-PCR

To validate the authenticity of mRNA sequencing results, seven genes related to immune defense were chosen for the qRT-PCR analysis (four up regulated genes and three down regulated genes), including trypsin-like, T-cell leukemia homeobox protein 3-like, C-type lectin 3, Toll interacting protein, caspase-1-like, glutathione peroxidase, and heat shock protein 90, aiming to investigate the expression changes of these genes after DIV1 infection. In addition, to further determine the authenticity of the sequencing results, five genes were randomly selected for qPCR analysis (two up regulated genes and three down regulated genes), including Kazal-type serine proteinase inhibitor 1-F, Chitotriosidase-1, Siwi, Cathepsin L precursor, and Tetratricopeptide repeat protein 27-like. As shown in Figure 5D, the expression patterns of these tested genes were consistent between RNA-Seq and qPCR. The data showed that all the gene expression profiles derived from RNA-Seq were reliable and confirmed the expression changes of these genes in response to DIV1 infection.

### 3.4. Correlations between altered intestine microbial and immunity in shrimp

**Figure 6:**
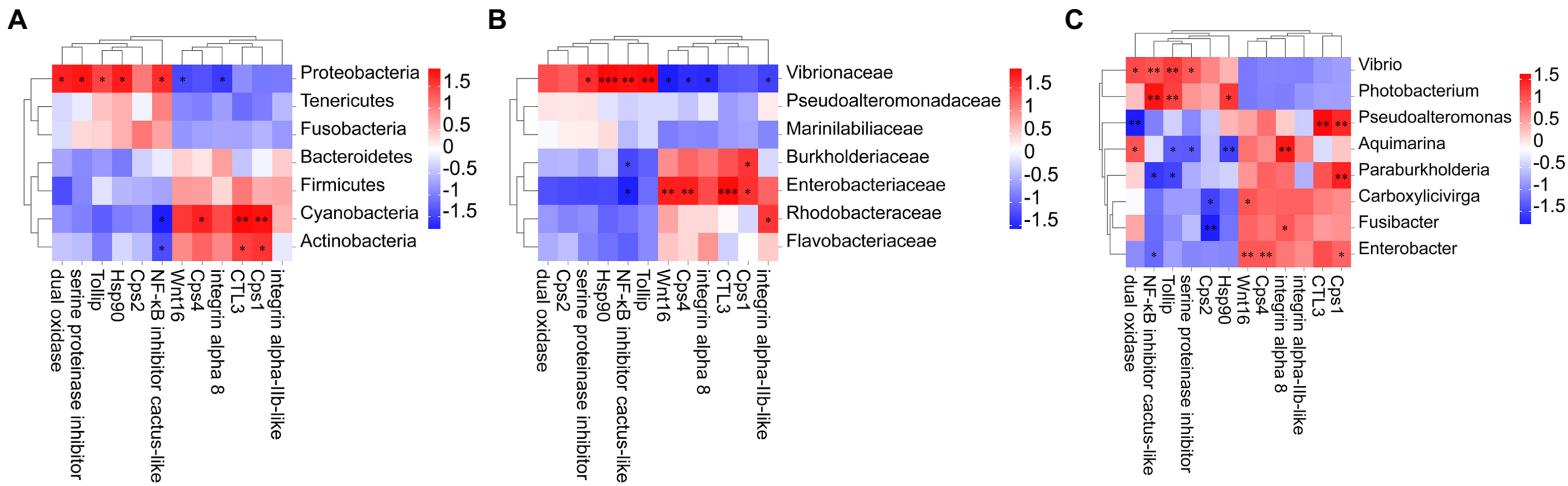
Heat map of the correlation between host intestinal bacteria under DIV1 infection at the phylum **(A)**, family **(B)** genus, and **(C)** classification levels and immune-related DEGs. Different colours indicate correlation coefficients. Red indicates positive correlations, and blue indicates negative correlations. * indicates significant differences (*p < 0.05; **p < 0.01; ***p < 0.001). Cps1, Caspase 1; Ctl3, C-type lectin 3; Cps4, Caspase 4; Tollip, Toll interacting protein; Hsp90, heat shock protein 90; Cps2, Caspase 2.

To reveal correlations between changes in intestinal microorganisms and intestinal immune-related DEGs, heat maps at the phylum, family, and genus level were generated using Pearson correlation analysis (Figure 6). At the phylum classification level, the abundance of Proteobacteria is positively correlated with dual oxidase, serine proteinase inhibitor, Tollip, Hsp90, and NF-κB inhibitor cactus-like, and negatively correlated with Wnt16 and integrin alpha 8. Cyanobacteria and Actinobacteria were negatively correlated with NF-κB inhibitor cactus-like and positively correlated with Ctl3 and caspase-1-like. Notably, *Photobacterium* and *Vibrio* in the Vibrionaceae, which were dominant in the DIV1-infected group, were positively correlated with Tollip and NF-κB inhibitor cactus-like. In contrast, *Aquimarina*, dominant in the PBS group, is negatively correlated with Tollip and NF-κB inhibitor cactus-like.

## 4. Discussion

Revealing that autoimmune regulation and intestine microbiota modulation are essential for enhancing disease resistance in shrimp. This study investigated the mechanism of interaction between the immune response and intestine microbes in *M. ensis* infected with DIV1. DIV1 infection resulted in a significant increase in the abundance of opportunistic pathogens such as *Vibrio* and *Photobacterium*, which may have induced the initiation of the melanization cascade. Antiviral-related pathways in the intestine were significantly activated following the DIV1 challenge. *M. ensis* combats DIV1 infection by enhancing the expression of some immune-related genes. Notably, DIV1 infection could increased Tollip and NF-κB inhibitor cactus-like expression through the expansion of *Vibrio* and *Photobacterium*, which may have limited the TLR-mediated immune response and ultimately led to further DIV1 infection. It is speculated that the virus could inhibit the immune response of the host by regulating the composition of the host microorganism, creating favorable conditions for the immune escape of the virus. This report, analysing the mechanism of the intestinal response to a DIV1 challenge from the perspective of molecular and microflora, enables a better understanding of the intestinal immune mechanism of *M. ensis* against DIV1 infection for the first time.

*M. ensis* infected with DIV1 showed apparent disease symptoms, including black body, soft shell, red stomach, empty intestine and atrophy of the hepatopancreas with yellowing. This is similar to the previous symptoms of DIV1 infection in *P. monodon*, *M. rosenbergii*, *L. vannamei,* and *M. japonicus* (Qiu et al., 2019; Liao X. et al., 2020; He et al., 2021a,b). Additionally, different shrimps species seem to have different pathological characteristics following infection with DIV1. For example, some *L. vannamei* and *P. monodon* have a black edge of the abdominal shell after infection with DIV1 (Liao X. et al., 2020; He et al., 2021a), and *M. rosenbergii* infected with DIV1 have a white triangular area at the base of the frontal horn (Qiu et al., 2019). The body color of *M. japonicus* turned red after infection with DIV1 (He et al., 2021b), while the body color of *P. monodon* turned black (He et al., 2021a). In the present study, some *M. ensis* had symptoms of black gill in addition to the black edge of the abdominal shell following DIV1 infection. These symptoms can be used as basic symptoms of DIV1 infection in shrimp, enabling the preliminary, visual assessment of whether DIV1 infection occurs in shrimp farming. LC_50_ is an important means of assessing the virulence of DIV1 and has been used in studies of DIV1 infection in *L. vannamei*, *P. monodon,* and *M. japonicus*. Among them, the LC_50_ is 3.91 × 10^7^ at 24 hpi in *L. vannamei*, 5.96 × 10^8^ at 44 hpi in *P. monodon* and 2.64 × 10^9^ at 36 hpi in *M. japonicus*, respectively (Liao X. et al., 2020; He et al., 2021a,b). In the current study, the LC_50_ results of DIV1 of *M. ensis* were different from those of other shrimp, which may be caused by the different types of tolerance in different shrimps to the pathogen.

Intestinal microflora’s normal structure and function are essential for maintaining intestinal homeostasis in shrimp (Dhar and Mohanty, 2020). The bacterial diversity was closely related with shrimp disease (Xiong et al., 2015). In the present study, the diversity of intestine microbiota decreased in *M. ensis* infected with DIV1, which could be attributed to viral infection weakened the ability of shrimp intestine to select microorganisms (Xiong et al., 2017). Furthermore, intestinal bacterial community compositions varied dramatically between the healthy and diseased *M. ensis*. At the phylum level, Proteobacteria, considered to be an opportunistic pathogen (Shin et al., 2015), significantly increased in shrimp infected with DIV1, while Bacteroidetes, Firmicutes, and Actinobacteria, which were functional bacteria related to host-health, were significantly decreased (Turnbaugh et al., 2009; Wang A. et al., 2019). At the level of family classification, the relative abundance of vibrionaceae in intestine of DIV1-infected shrimps was significantly increased compared to healthy shrimps, which was mainly reflected in a significant increase in the relative abundance of *Photobacterium* and *Vibrio*. Toxicity tests show that *Photobacterium* was associated with muscle necrosis and hepatopancreas lesions in *L. vannamei* (Singaravel et al., 2020). *Vibrio* is one of the most abundant genera in the shrimp intestine (Huang F. et al., 2018; Fan et al., 2019). Vibrio’s overabundance could change the shrimp’s health status and increase the risk of disease outbreaks (Xiong et al., 2017; Huang X. et al., 2018). Therefore, the increased relative abundance of *Photobacterium* and *Vibrio* in *M. ensis* may increase the risk of secondary bacterial infection. PICRUSt functional prediction results revealed that the mean abundance of “Bacterial chemotaxis” and “Flagellar assembly,” which are important features of pathogen colonization and infection (Freter, 1981; Freter and O'Brien, 1981), was significantly increased after *M. ensis* infection with DIV1 (*p* < 0.01). Furthermore, the relative abundance of “Biofilm formation-Vibrio cholerae” was significantly increased under DIV1 infection (*p* < 0.01), further suggesting that DIV1 infection may lead to the occurrence of secondary bacterial infection. In addition, The mean abundance of several well-known metabolism-related pathways were significantly increased in the intestine of *M. ensis* infected with DIV1, including Alanine, aspartate and glutamate metabolism, Lipoic acid metabolism, Pyruvate metabolism, Nicotinate and nicotinamide metabolism, and Pyrimidine metabolism, which could be attributed to the bioenergetic and biosynthetic requirements for DIV1 replication in shrimp (Chen et al., 2011).

Multiple potential immune-related genes were screened from the DIV1-infected group and the PBS group by comparative transcriptomic analysis, including Wnt16, Ctl3, Hsp90, Tollip, and NF-κB inhibitor cactus-like. C-type lectins are one of the PRRs in invertebrates, which play a central role in innate immunity for shrimp (Thiel and Gadjeva, 2009). When infected by WSSV, C-type lectins can inhibit the pathological effects of WSSV in hemocytes by combining with several structural proteins of WSSV (Zhao et al., 2009). Hsp90 is a protective protein synthesized in large amounts to help each cell maintain regular physiological activity when the host’s living conditions suffer mutation (Sato et al., 2000). As one of the Wnt family members, Wnt16 is involved in the immune response to pathogen infection (Zhu and Zhang, 2013). In our research, the expression level of Ctl3, Hsp90 and Wnt16 were significantly upregulated, implying that these genes may involved in the defense mechanism of shrimp anti-DIV1. GO enrichment analysis showed GO terms associated with virus invasion, replication and host antiviral infection were activated after DIV1 infection, including ncRNA metabolic process and ncRNA processing. ncRNA, especially lncRNA, has been shown to inhibit viral infection or stimulate the host antiviral immune response (Wang, 2019). Multiple lncRNAs were co-expressed with immune-related genes to regulate the immune defense of blood cells during *Spiroplasma Eriocheiris* infections in *L. vannamei* (Ren et al., 2020). Liu et al. (2019) identified 163 immune-related lncRNAs by transcriptome assembly involved in the immune response to large yellow croaker (*Larimichthys crocea*) infection with *Vibrio parahaemolyticus*. The top 20 KEGG pathways affected by DIV1 infection, Ascorbate and aldarate metabolism and Arachidonic acid metabolism were significantly activated. Ascorbic acid (Vitamin C (VC)) improves survival and development rates and also helps to enhance the immune system (Tewary and Patra, 2008). Dietary supplementation of VC could enhance immunoglobulin and prophenoloxidase activity in shrimp serum (Wang et al., 2002). The latter is the shrimp’s key enzyme inactivating melanization (Tassanakajon et al., 2018). Additionally, dietary Arachidonic acid (ARA) can increase the activities of oxide dismutase and catalase, improve the serum lysozyme activity, and the disease resistance of shrimp (Duan et al., 2022). In this study, Ascorbate and aldarate metabolism and Arachidonic acid metabolism pathways were significantly different following DIV1 infection, indicating that *M. ensis* could enhance immune defense by regulating the metabolism of VC and ARA. Finally, several well-known antiviral immune-related pathways were also activated, including the Wnt signaling pathway, p53 signaling pathway, C-type lectin receptor signaling pathway, Toll and Imd signaling pathway, NOD-like receptor signaling pathway, and PI3K-Akt signaling pathway. These pathways are all activated in DIV1-infected *L. vannamei*, *M. japonicus*, *F. merguiensis*, and *P. monodon* (Liao X.Z. et al., 2020; Liao X. et al., 2020; He et al., 2021a,b). Therefore, they may play an anti-virus role during DIV1 infections in *M. ensis*.

Intestine microbiota dysbiosis initiated by diseases may further influence host-regulating immune functions (Pérez et al., 2010). In recent years, TLRs-mediated signaling cascade have received increasing attentions due to their role in innate immunity and disease resistance. Invertebrate host cells have been shown to recognize PAMPs on microbial pathogens through TLRs, and activate innate immune responses (Lu et al., 2013; Li et al., 2019; Dhar and Mohanty, 2020). Tollip and inhibitor of NF-κB (IκB) are potential negative regulators of TLR-signaling cascade in shrimp (Lu et al., 2013). Studies have shown that the negative regulation of TLR signaling by Tollip and IκB may help to limit the production of proinflammatory mediators during inflammation and infection (Zhang et al., 2002; Li et al., 2019). In the present study, NF-κB inhibitor cactus-like and Tollip were significantly upregulated after *M. ensis* infected DIV1, which may limit the TLR-mediated immune response. Interestingly, the correlation analysis between intestinal microbial variation and host immunity showed that an elevated abundance of *Photobacterium* and *Vibrio* could increase expression of Tollip and NF-κB inhibitor cactus-like, suggesting that the expansion of *Photobacterium* and *Vibrio* in DIV1 infection could be a key factor to limit TLR-mediated immune response, which may ultimately lead to further infection of DIV1. Melanization is a phenomenon of melanin deposition in the injured site of crustaceans when they are attacked by pathogens. It has been found that melanization cascade plays an immunological role in the host’s resistance to bacterial, fungal and viral infections (Tassanakajon et al., 2018). Upon pathogen invasion, the ProPO system uses the binding of specific molecular PRRs to corresponding microbial cell wall components to activates the melanization cascade (Amparyup et al., 2013). In this study, some *M. ensis* infected with DIV1 showed blackbody symptoms, which may be the initiation of melanization induced by the expansion of *Vibrio* and *Photobacterium* in DIV1 infection. In addition, Wang et al. (2020) isolated three subspecies of *Photobacterium* from *L. vannamei* with black gill disease, and demonstrated that the strain was the pathogenic bacteria *L. vannamei* using LC_50_ tests. It is speculated that the black gill symptom of *M. ensis* infected with DIV1 may be caused by the increased relative abundance of *Photobacterium*.

## 5. Conclusion

In conclusion, we determined the LC_50_ values of DIV1-infected *M. ensis*, and the host-intestinal microbiota interactions and responses to infection with DIV1 were also investigated. DIV1 infection decreased bacteria diversity and changed the composition of the microbial in the shrimp intestine. Several antiviral-pathways in intestine were significantly activated. Shrimp combats DIV1 infection by enhancing the expression of some immune-related genes. Futhermore, the expansion of harmful bacteria (*Vibrio* and *Photobacterium*) during DIV1 infection may limit the TLR-mediated immune response that ultimately leads to DIV1 infection. Further studies will focus on how to promote the shrimp intestine microbiota to increase the transcripts of TLRs, thereby improving the resistance to DIV1 infection.

## Data availability statement

The datasets presented in this study can be found in online repositories. The names of the repository/repositories and accession number(s) can be found at: https://www.ncbi.nlm.nih.gov/, SRP393433 https://www.ncbi.nlm.nih.gov/, SRP394201.

## Ethics statement

The animal study was reviewed and approved by The Ethics Review Board of the Institutional Animal Care and Use Committee at Guangdong Ocean University.

## Author contributions

ML and CS conceived and designed the study. ML, XinL, JicZ, ZH, JinZ, and TW contributed to the conduct of experiment, sample collection, data collection, and analysis. The first draft of the manuscript was written by ML and revised by XuzL. CS performed the final review and editing and contributed to the project administration and funding acquisition. All authors contributed to the article and approved the submitted version.

## Funding

This research was funded by the key research and development projects in Guangdong Province (grant no. 2020B0202010009), and the project of the innovation team for the innovation and utilization of Economic Animal Germplasm in the South China Sea (grant no. 2021KCXTD026).

## Conflict of interest

The authors declare that they have no known competing financial interests or personal relationships that could have appeared to influence the work reported in this paper.

## Publisher’s note

All claims expressed in this article are solely those of the authors and do not necessarily represent those of their affiliated organizations, or those of the publisher, the editors and the reviewers. Any product that may be evaluated in this article, or claim that may be made by its manufacturer, is not guaranteed or endorsed by the publisher.

## Footnotes

^1^ http://www.ncbi.nlm.nih.gov

^2^ http://www.expasy.ch/sprot

^3^ http://www.geneontology.org/

^4^ http://www.ncbi.nlm.nih.gov/pubmed/14759257

^5^ http://www.genome.jp/kegg/

^6^ http://www.ncbi.nlm.nih.gov/Traces/sra

## References

[ref1] Amparyup P. Charoensapsri W. Tassanakajon A. (2013). Prophenoloxidase system and its role in shrimp immune responses against major pathogens. Fish Shellfish Immunol. 34, 990–1001. doi: 10.1016/j.fsi.2012.08.019, PMID: 22960099

[ref2] Bliss C. I. (1939). The toxicity of poisons applied jointly1. Ann. Appl. Biol. 26, 585–615. doi: 10.1111/j.1744-7348.1939.tb06990.x

[ref3] Bokulich N. A. Subramanian S. Faith J. J. Gevers D. Gordon J. I. Knight R.. (2013). Quality-filtering vastly improves diversity estimates from Illumina amplicon sequencing. Nat. Methods 10, 57–59. doi: 10.1038/nmeth.2276, PMID: 23202435 PMC3531572

[ref4] Chen I.-T. Aoki T. Huang Y.-T. Hirono I. Chen T.-C. Huang J.-Y.. (2011). White spot syndrome virus induces metabolic changes resembling the Warburg effect in shrimp Hemocytes in the early stage of infection. J. Virol. 85, 12919–12928. doi: 10.1128/JVI.05385-11, PMID: 21976644 PMC3233138

[ref5] Chen X. Qiu L. Wang H. Zou P. Dong X. Li F.. (2019). Susceptibility of *Exopalaemon carinicauda* to the infection with shrimp hemocyte iridescent virus (SHIV 20141215), a strain of decapod iridescent virus 1 (DIV1). Viruses 11:387. doi: 10.3390/v11040387 31027252 PMC6520858

[ref6] Clemente J. C. Ursell L. K. Parfrey L. W. Knight R. (2012). The impact of the gut microbiota on human health: an integrative view. Cells 148, 1258–1270. doi: 10.1016/j.cell.2012.01.035, PMID: 22424233 PMC5050011

[ref7] Cui J. Wu L. Chan S.-M. Chu K. H. (2013). cDNA cloning and mRNA expression of retinoid-X-receptor in the ovary of the shrimp *Metapenaeus ensis*. Mol. Biol. Rep. 40, 6233–6244. doi: 10.1007/s11033-013-2735-8, PMID: 24091942

[ref8] Dhar D. Mohanty A. (2020). Gut microbiota and Covid-19-possible link and implications. Virus Res. 285:198018. doi: 10.1016/j.virusres.2020.198018, PMID: 32430279 PMC7217790

[ref9] Ding Z. F. Cao M. J. Zhu X. S. Xu G. H. Wang R. L. (2017). Changes in the gut microbiome of the Chinese mitten crab (Eriocheir sinensis) in response to white spot syndrome virus (WSSV) infection. J. Fish Dis. 40, 1561–1571. doi: 10.1111/jfd.12624, PMID: 28429823

[ref10] Duan Y. Liu Q. Wang Y. Zhang J. Xiong D. (2018). Impairment of the intestine barrier function in *Litopenaeus vannamei* exposed to ammonia and nitrite stress. Fish Shellfish Immunol. 78, 279–288. doi: 10.1016/j.fsi.2018.04.050, PMID: 29709590

[ref11] Duan Y. Lu Z. Zeng S. Dan X. Zhang J. Li Y. (2022). Effects of dietary arachidonic acid on growth, immunity and intestinal microbiota of *Litopenaeus vannamei* under microcystin-LR stress. Aquaculture 549:737780. doi: 10.1016/j.aquaculture.2021.737780

[ref13] Edgar R. C. Haas B. J. Clemente J. C. Quince C. Knight R. (2011). UCHIME improves sensitivity and speed of chimera detection. Bioinformatics 27, 2194–2200. doi: 10.1093/bioinformatics/btr381, PMID: 21700674 PMC3150044

[ref14] Fan L. Wang Z. Chen M. Qu Y. Li J. Zhou A.. (2019). Microbiota comparison of Pacific white shrimp intestine and sediment at freshwater and marine cultured environment. Sci. Total Environ. 657, 1194–1204. doi: 10.1016/j.scitotenv.2018.12.069, PMID: 30677886

[ref15] Freter R. (1981). Mechanisms of association of bacteria with mucosal surfaces. Ciba Found. Symp. 80, 36–55. PMID: 7021088 10.1002/9780470720639.ch4

[ref16] Freter R. O'Brien P. C. (1981). Role of chemotaxis in the association of motile bacteria with intestinal mucosa: chemotactic responses of vibrio cholerae and description of motile nonchemotactic mutants. Infect. Immun. 34, 215–221. doi: 10.1128/iai.34.1.215-221.1981, PMID: 7298183 PMC350845

[ref17] Grabherr M. G. Haas B. J. Yassour M. Levin J. Z. Thompson D. A. Amit I.. (2011). Full-length transcriptome assembly from RNA-Seq data without a reference genome. Nat. Biotechnol. 29, 644–652. doi: 10.1038/nbt.1883, PMID: 21572440 PMC3571712

[ref18] Guo M. Wu F. Hao G. Qi Q. Li R. Li N.. (2017). Bacillus subtilis improves immunity and disease resistance in rabbits. Front. Immunol. 8:354. doi: 10.3389/fimmu.2017.00354, PMID: 28424690 PMC5372816

[ref20] He Z. Chen X. Zhao J. Hou D. Fu Z. Zhong Y.. (2021a). Establishment of infection mode and *Penaeus monodon* hemocytes transcriptomics analysis under decapod iridescent virus 1 (DIV1) challenge. Aquaculture 542:736816. doi: 10.1016/j.aquaculture.2021.736816

[ref21] He Y. Chi S. Tan B. Zhang H. Dong X. Yang Q.. (2017). Effect of yeast culture on intestinal microbiota of *Litopenaeus vannamei*. J Guangdong Ocean Univ. 37, 21–27.

[ref22] He Z. Zhao J. Chen X. Liao M. Xue Y. Zhou J.. (2021b). The molecular mechanism of Hemocyte immune response in *Marsupenaeus japonicus* infected with decapod iridescent virus 1. Front. Microbiol. 12:710845. doi: 10.3389/fmicb.2021.710845 34512588 PMC8427283

[ref23] He Z. Zhong Y. Hou D. Hu X. Fu Z. Liu L.. (2022). Integrated analysis of mRNA-Seq and MiRNA-Seq reveals the molecular mechanism of the intestinal immune response in *Marsupenaeus japonicus* under decapod iridescent virus 1 infection. Front. Immunol. 12:807093. doi: 10.3389/fimmu.2021.807093, PMID: 35116034 PMC8804360

[ref24] Huang F. Pan L. Song M. Tian C. Gao S. (2018). Microbiota assemblages of water, sediment, and intestine and their associations with environmental factors and shrimp physiological health. Appl. Microbiol. Biotechnol. 102, 8585–8598. doi: 10.1007/s00253-018-9229-5, PMID: 30039332

[ref25] Huang X. Wen C. Liang H. Hong J. Xue M. (2018). Comparison of bacterial community structure in larval rearing water between healthy and diseased *Litopenaeus vannamei* mysis. J Guangdong Ocean Univ. 38, 27–34.

[ref26] Jia W. Xie G. Jia W. (2018). Bile acid-microbiota crosstalk in gastrointestinal inflammation and carcinogenesis. Nat. Rev. Gastroenterol. Hepatol. 15, 111–128. doi: 10.1038/nrgastro.2017.119, PMID: 29018272 PMC5899973

[ref27] Khan I. Bai Y. Zha L. Ullah N. Ullah H. Shah S. R. H.. (2021). Mechanism of the gut microbiota colonization resistance and enteric pathogen infection. Front. Cell. Infect. Microbiol. 11:716299. doi: 10.3389/fcimb.2021.716299 35004340 PMC8733563

[ref28] Langille M. G. I. Zaneveld J. Caporaso J. G. McDonald D. Knights D. Reyes J. A.. (2013). Predictive functional profiling of microbial communities using 16S rRNA marker gene sequences. Nat. Biotechnol. 31, 814–821. doi: 10.1038/nbt.2676, PMID: 23975157 PMC3819121

[ref29] Li C. Wang S. He J. (2019). The two NF-κB pathways regulating bacterial and WSSV infection of shrimp. Front. Immunol. 10:1785. doi: 10.3389/fimmu.2019.01785, PMID: 31417561 PMC6683665

[ref30] Liao X. He J. Li C. (2022). Decapod iridescent virus 1: an emerging viral pathogen in aquaculture. Rev. Aquac. 14, 1779–1789. doi: 10.1111/raq.12672

[ref31] Liao X. Wang C. Wang B. Qin H. Hu S. Wang P.. (2020). Comparative transcriptome analysis of *Litopenaeus vannamei* reveals that Triosephosphate isomerase-like genes play an important role during decapod iridescent virus 1 infection. Front. Immunol. 11:1904. doi: 10.3389/fimmu.2020.01904, PMID: 32983114 PMC7485339

[ref32] Liao X.-Z. Wang C.-G. Wang B. Qin H.-P. Hu S.-K. Zhao J.-C.. (2020). Research into the hemocyte immune response of *Fenneropenaeus merguiensis* under decapod iridescent virus 1 (DIV1) challenge using transcriptome analysis. Fish Shellfish Immunol. 104, 8–17. doi: 10.1016/j.fsi.2020.05.053 32473357

[ref33] Liu X. Li W. Jiang L. Lü Z. Liu M. Gong L.. (2019). Immunity-associated long non-coding RNA and expression in response to bacterial infection in large yellow croaker (Larimichthys crocea). Fish Shellfish Immunol. 94, 634–642. doi: 10.1016/j.fsi.2019.09.015, PMID: 31533082

[ref35] Livak K. J. Schmittgen D. T. (2001). Analysis of relative gene expression data using real-time quantitative PCR and the 2(-Delta Delta C(T)) method. Methods 25, 402–408. doi: 10.1006/meth.2001.1262 11846609

[ref36] Love M. I. Huber W. Anders S. (2014). Moderated estimation of fold change and dispersion for RNA-seq data with DESeq2. Genome Biol. 15:550. doi: 10.1186/s13059-014-0550-8, PMID: 25516281 PMC4302049

[ref37] Lu Y. Li C. Wang D. Su X. Jin C. Li Y.. (2013). Characterization of two negative regulators of the toll-like receptor pathway in Apostichopus japonicus: inhibitor of NF-κB and toll-interacting protein. Fish Shellfish Immunol. 35, 1663–1669. doi: 10.1016/j.fsi.2013.08.014, PMID: 23978566

[ref39] Negi S. Das D. K. Pahari S. Nadeem S. Agrewala J. N. (2019b). Potential role of gut microbiota in induction and regulation of innate immune memory. Front. Immunol. 10:2441. doi: 10.3389/fimmu.2019.02441, PMID: 31749793 PMC6842962

[ref40] Negi S. Pahari S. Bashir H. Agrewala J. N. (2019a). Gut microbiota regulates Mincle mediated activation of lung dendritic cells to protect against mycobacterium tuberculosis. Front. Immunol. 10:1142. doi: 10.3389/fimmu.2019.01142, PMID: 31231363 PMC6558411

[ref42] Pérez T. Balcázar J. L. Ruiz-Zarzuela I. Halaihel N. Vendrell D. Blas L. D.. (2010). Host-microbiota interactions within the fish intestinal ecosystem. Mucosal Immunol. 3, 355–360. doi: 10.1038/mi.2010.12 20237466

[ref43] Qiu L. Chen M.-M. Wan X.-Y. Li C. Zhang Q.-L. Wang R.-Y.. (2017). Characterization of a new member of Iridoviridae, shrimp hemocyte iridescent virus (SHIV), found in white leg shrimp (*Litopenaeus vannamei*). Sci. Rep. 7:11834. doi: 10.1038/s41598-017-10738-8, PMID: 28928367 PMC5605518

[ref45] Qiu L. Chen X. Zhao R.-H. Li C. Gao W. Zhang Q.-L.. (2019). Description of a natural infection with decapod iridescent virus 1 in farmed Giant freshwater prawn, *Macrobrachium rosenbergii*. Viruses 11:354. doi: 10.3390/v11040354 30999644 PMC6521035

[ref46] Ramírez C. Coronado J. Silva A. Romero J. (2018). Cetobacterium is a major component of the microbiome of Giant Amazonian fish (Arapaima gigas) in Ecuador. Animals 8:189. doi: 10.3390/ani8110189, PMID: 30352962 PMC6262583

[ref47] Ren Y. Li J. Guo L. Liu J. N. Wan H. Meng Q.. (2020). Full-length transcriptome and long non-coding RNA profiling of whiteleg shrimp Penaeus vannamei hemocytes in response to Spiroplasma eriocheiris infection. Fish Shellfish Immunol. 106, 876–886. doi: 10.1016/j.fsi.2020.06.057, PMID: 32800983

[ref48] Rungrassamee W. Klanchui A. Maibunkaew S. Karoonuthaisiri N. (2016). Bacterial dynamics in intestines of the black tiger shrimp and the Pacific white shrimp during Vibrio harveyi exposure. J. Invertebr. Pathol. 133, 12–19. doi: 10.1016/j.jip.2015.11.004, PMID: 26585302

[ref49] Sato S. Fujita N. Tsuruo T. (2000). Modulation of Akt kinase activity by binding to Hsp90. Proc. Natl. Acad. Sci. U. S. A. 97, 10832–10837. doi: 10.1073/pnas.170276797, PMID: 10995457 PMC27109

[ref51] Shin N.-R. Whon T. W. Bae J.-W. (2015). Proteobacteria: microbial signature of dysbiosis in gut microbiota. Trends Biotechnol. 33, 496–503. doi: 10.1016/j.tibtech.2015.06.011 26210164

[ref52] Siddique M. A. Haque M. I.-M. Sanyal S. K. Hossain A. Nandi S. P. Alam A. S. M. R. U.. (2018). Circulatory white spot syndrome virus in south-west region of Bangladesh from 2014 to 2017: molecular characterization and genetic variation. AMB Express 8:25. doi: 10.1186/s13568-018-0553-z, PMID: 29460184 PMC5818386

[ref53] Simão F. A. Waterhouse R. M. Ioannidis P. Kriventseva E. V. Zdobnov E. M. (2015). BUSCO: assessing genome assembly and annotation completeness with single-copy orthologs. Bioinformatics 31, 3210–3212. doi: 10.1093/bioinformatics/btv351 26059717

[ref54] Singaravel V. Gopalakrishnan A. Dewangan N. K. Kannan D. Shettu N. Martin G. G. (2020). *Photobacterium damselae* subsp. *damselae* associated with bacterial myonecrosis and hepatopancreatic necrosis in broodstock Pacific white leg shrimp, *Litopenaeus vannamei* (Boone, 1931). Aquac. Int. 28, 1593–1608. doi: 10.1007/s10499-020-00545-w

[ref56] Sun Y. Li F. Xiang J. (2013). Analysis on the dynamic changes of the amount of WSSV in Chinese shrimp Fenneropenaeus chinensis during infection. Aquaculture 376, 124–132. doi: 10.1016/j.aquaculture.2012.11.014

[ref58] Tang K. F. J. Navarro S. A. Lightner D. V. (2007). PCR assay for discriminating between infectious hypodermal and hematopoietic necrosis virus (IHHNV) and virus-related sequences in the genome of *Penaeus monodon*. Dis. Aquat. Org. 74, 165–170. doi: 10.3354/dao074165, PMID: 17432046

[ref59] Tassanakajon A. Rimphanitchayakit V. Visetnan S. Amparyup P. Somboonwiwat K. Charoensapsri W.. (2018). Shrimp humoral responses against pathogens: antimicrobial peptides and melanization. Dev. Comp. Immunol. 80, 81–93. doi: 10.1016/j.dci.2017.05.009, PMID: 28501515

[ref60] Tewary A. Patra B. C. (2008). Use of vitamin C as an immunostimulant. Effect on growth, nutritional quality, and immune response of *Labeo rohita* (ham.). Fish Physiol. Biochem. 34, 251–259. doi: 10.1007/s10695-007-9184-z, PMID: 18665463

[ref61] Thiel S. Gadjeva M. (2009). Humoral pattern recognition molecules: Mannan-binding lectin and Ficolins. Target Pattern Recognit. Innate Immun. 653, 58–73. doi: 10.1007/978-1-4419-0901-5_5, PMID: 19799112

[ref62] Turnbaugh P. J. Hamady M. Yatsunenko T. Cantarel B. L. Duncan A. Ley R. E.. (2009). A core gut microbiome in obese and lean twins. Nature 457, 480–484. doi: 10.1038/nature07540, PMID: 19043404 PMC2677729

[ref63] Wang P. (2019). The opening of Pandora's box: an emerging role of long noncoding RNA in viral infections. Front. Immunol. 9:3138. doi: 10.3389/fimmu.2018.03138, PMID: 30740112 PMC6355698

[ref65] Wang J. Huang Y. Xu K. Zhang X. Sun H. Fan L.. (2019). White spot syndrome virus (WSSV) infection impacts intestinal microbiota composition and function in *Litopenaeus vannamei*. Fish Shellfish Immunol. 84, 130–137. doi: 10.1016/j.fsi.2018.09.076, PMID: 30278220

[ref66] Wang W. Li A. Cheung S. (2002). Effects of dietary vitamin C on the immune function of shrimps, *Penaeus chinensis*. J. Ocean Univ. Qingdao. 1, 50–54. doi: 10.1007/s11802-002-0030-8

[ref67] Wang A. Ran C. Wang Y. Zhang Z. Ding Q. Yang Y.. (2019). Use of probiotics in aquaculture of China-a review of the past decade. Fish Shellfish Immunol. 86, 734–755. doi: 10.1016/j.fsi.2018.12.026, PMID: 30553887

[ref68] Wang Z. Shi C. Wang H. Wan X. Zhang Q. Song X.. (2020). A novel research on isolation and characterization of *Photobacterium damselae* subsp. *damselae* from Pacific white shrimp, *Penaeus vannamei*, displaying black gill disease cultured in China. J. Fish Dis. 43, 551–559. doi: 10.1111/jfd.13153, PMID: 32196691

[ref69] Wu Y. Li R. Shen G. Huang F. Yang Q. Tan B.. (2021). Effects of dietary small peptides on growth, antioxidant capacity, nonspecific immunity and lngut microflora structure of *Litopenaeus vannamei*. J Guangdong Ocean Univ. 41, 1–9.

[ref70] Xiao F. Liao L. Xu Q. He Z. Xiao T. Wang J.. (2021). Host-microbiota interactions and responses to grass carp reovirus infection in *Ctenopharyngodon idellus*. Environ. Microbiol. 23, 431–447. doi: 10.1111/1462-2920.15330, PMID: 33201573

[ref71] Xiong J. Wang K. Wu J. Qiuqian L. Yang K. Qian Y.. (2015). Changes in intestinal bacterial communities are closely associated with shrimp disease severity. Appl. Microbiol. Biotechnol. 99, 6911–6919. doi: 10.1007/s00253-015-6632-z, PMID: 25947250

[ref72] Xiong J. Zhu J. Dai W. Dong C. Qiu Q. Li C. (2017). Integrating gut microbiota immaturity and disease-discriminatory taxa to diagnose the initiation and severity of shrimp disease. Environ. Microbiol. 19, 1490–1501. doi: 10.1111/1462-2920.13701, PMID: 28205371

[ref73] Xu L. Wang T. Li F. Yang F. (2016). Isolation and preliminary characterization of a new pathogenic iridovirus from redclaw crayfish *Cherax quadricarinatus*. Dis. Aquat. Org. 120, 17–26. doi: 10.3354/dao03007, PMID: 27304867

[ref74] Yu W. Wu J.-H. Zhang J. Yang W. Chen J. Xiong J. (2018). A meta-analysis reveals universal gut bacterial signatures for diagnosing the incidence of shrimp disease. FEMS Microbiol. Ecol. 94. doi: 10.1093/femsec/fiy147, PMID: 30124839

[ref75] Zhang G. Ghosh S. Footnotes S. (2002). Negative regulation of toll-like receptor-mediated signaling by Tollip. J. Biol. Chem. 277, 7059–7065. doi: 10.1074/jbc.M109537200, PMID: 11751856

[ref77] Zhao Z.-Y. Yin Z.-X. Xu X.-P. Weng S.-P. Rao X.-Y. Dai Z.-X.. (2009). A novel C-type lectin from the shrimp *Litopenaeus vannamei* possesses anti-white spot syndrome virus activity. J. Virol. 83, 347–356. doi: 10.1128/JVI.00707-08, PMID: 18945787 PMC2612311

[ref78] Zhu F. Zhang X. (2013). The Wnt signaling pathway is involved in the regulation of phagocytosis of virus in drosophila. Sci. Rep. 3:2069. doi: 10.1038/srep02069, PMID: 23797713 PMC3691566

