# Supplementary material for: Host-microbiota interactions and responses of Metapenaeus ensis infected with decapod iridescent virus 1
Source: Front Microbiol. 2023 Jan 13;13:1097931. doi: 10.3389/fmicb.2022.1097931 (PMC9880205; doi:10.3389/fmicb.2022.1097931)
Supplement: Supplementary file 1 [file Data_Sheet_1.PDF]

Supplementary materials

Host-microbiota interactions and responses of *Metapenaeus ensis* infected with decapod iridescent virus 1

Minze Liao[1†], Xuzheng Liao[2†], Xinxin Long[1], Jichen Zhao[3], Zihao He[1], Jingyue Zhang[1], Tingfen Wu[1], Chengbo Sun[1, 4, 5,*]

[1] College of Fisheries, Guangdong Ocean University, Zhanjiang, Guangdong, China

[2] School of Marine Sciences, Sun Yat-sen University, Guangzhou, People's Republic of China

[3] Institute of Animal Science, Guangdong Academy of Agricultural Sciences, Key Laboratory of Animal Nutrition and Feed Science in South China, Ministry of Agriculture and Rural Affairs, Guangdong Provincial Key Laboratory of Animal Breeding and Nutrition, Guangzhou 510640, China

[4] Guangdong Provincial Key Laboratory of Pathogenic Biology and Epidemiology for Aquatic Economic Animals, Zhanjiang, Guangdong, China

[5] Southern Marine Science and Engineering Guangdong Laboratory (Zhanjiang), Zhanjiang, Guangdong, China

[†] These authors have contributed equally to this work

[*] Corresponding author: suncb@gdou.edu.cn (C.B. Sun)

College of Fisheries, Guangdong Ocean University, 1 Haida Road, Zhanjiang 524088, PR China

Table S1

Primers used in the validation of gene expression.

No.	Primer names	Sequences(5′-3′)	References	Amplicon size
PCR				
1	WSSV-F	TGTGACCAAGACCATCGAAA	(Siddique et al., 2018)	643 bp
2	WSSV-R	CTTGATTTTGCCCAAGGTGT		
3	IHHNV-F	TCCAACACTTAGTCAAAACCAA	(Tang et al., 2007)	309 bp
4	IHHNV-R	TGTCTGCTACGATGATTATCCA		
5	DIV1-F	GGGCGGGAGATGGTGTTAGAT	(Qiu et al., 2017)	457 bp
6	DIV1-R	TCGTTTCGGTACGAAGATGTA		
qPCR				
7	qRT-DIV1-F	AATCCATGCAAGGTTCCTCAGG	(Qiu et al., 2020)	-
8	qRT-DIV1-R	CAATCAACATGTCGCGGTGAAC		
9	TaqMan probe	CCATACGTGCTCGCTCGGCTTCGG		
10	Kazal-type serine proteinase inhibitor 1-F	GCTCGCCGACTGTCTTAG	-	-
11	Kazal-type serine proteinase inhibitor 1-R	TCGCATGGGTCATCTTTC		
12	trypsin-like-F	ATCTCCACCAGGTCACGC	-	-
13	trypsin-like-R	GGGTCCACTTCCCATCCT		
14	T-cell leukemia homeobox protein 3-like-F	AAGAAGCCTCGCACCTCC	-	-
15	T-cell leukemia homeobox protein 3-like-R	TCGCTCGACGCCAAATAC		
16	Chitotriosidase-1-F	TCTCAGCGTTGGCGATTC	-	-
17	Chitotriosidase-1-R	CCTGTCCGACCTCATTGTTT		
18	Glutathione peroxidase-F	CGGACACTCACCTTACCACT	-	-
19	Glutathione peroxidase-R	AACGGGACCATCTACGACTT		
20	Caspase-1-like-F	TGAACTTGGCGAAGGATGA	-	-
21	Caspase-1-like-R	TGAACACGACGACCAGACA		
22	Siwi-F	TGTTCTTCTGGTAGGAGCCG	-	-
23	Siwi-R	TCCAAGCATTGATCCGTTT		
24	Cathepsin L precursor-F	CCTCCCATCCTTCCTTCCA	-	-
25	Cathepsin L precursor-R	CTTGCCGTCGTCTGTCTCG		
26	Heat shock protein 90-F	GAATACGACGGCAAACAACT	-	-
27	Heat shock protein 90-R	TCACAACCACCTTCTCCACT		
28	Tetratricopeptide repeat protein 27-like-F	GTTCGCCTACCAAGTGCC	-	-
29	Tetratricopeptide repeat	ATGATGTGGGTCGTGCTG		

	protein 27-like-R			
30	C-type lectin 3-F	CGTGGGCTCCTGCTTGTT	-	-
31	C-type lectin 3-R	TTCTGGGTCGGCGCTTCT		
32	Toll interacting protein-F	TGGCATCATCATCATCGG	-	-
33	Toll interacting protein-R	AGGGACAGGAGGGCAGTA		
34	EF1α-F	CGCCACCTGCTTCCTTCT	(Cui et al., 2013)	-
35	EF1α-R	TGCCACACTGCTCACATT		

Table S2

16S rRNA gene sequencing results of 10 samples from the DIV1-infected and PBS groups.

Sample name	Raw Reads	Clean Reads	Raw tags	Clean tags	Effective tags	Taxon tags	OTUs	N50	N90
DIV1-1	126,115	125,875	117,866	116,694	115,289	108,053	262	466	441
DIV1-2	135,015	134,765	126,880	125,879	123,641	112,970	301	466	441
DIV1-3	133,839	133,521	122,858	121,354	116,084	91,059	404	466	441
DIV1-4	130,483	130,239	122,791	121,815	120,256	112,723	256	466	449
DIV1-5	133,869	133,612	124,023	122,695	119,283	104,858	296	466	441
PBS-1	132,956	131,209	107,006	105,441	104,169	97,948	280	461	441
PBS-2	132,271	131,355	115,827	114,213	112,685	105,834	380	461	441
PBS-3	122,168	121,941	114,702	113,022	111,616	104,618	229	466	441
PBS-4	124,322	124,114	117,971	116,981	115,470	108,633	280	461	441
PBS-5	124,342	123,186	104,562	103,146	102,193	95,764	271	461	441

Table S3

Significant difference analysis of relative abundances of the top 10 dominant bacteria in DIV1-infected group and PBS group.

Taxonomy (%)	DIV1-infected group	PBS group	*p* value
**Phylum**			
Proteobacteria	79.11	60.77	0.0018
Bacteroidetes	9.48	17.67	0.0053
Firmicutes	1.71	7.92	0.0002
Cyanobacteria	1.67	4.06	0.0007
Fusobacteria	2.06	3.29	0.0334
Tenericutes	2.58	1.23	0.0004
Actinobacteria	1.32	2.05	0.0068
Patescibacteria	0.81	1.24	0.2613
Planctomycetes	0.83	0.83	0.9921
Chlamydiae	0.10	0.17	0.7139
**Family**			
Vibrionaceae	46.58	12.15	< 0.0001
Rhodobacteraceae	10.71	13.25	0.0073
Flavobacteriaceae	4.74	10.43	0.0001
Pseudoalteromonadaceae	6.58	7.19	0.0557
Burkholderiaceae	4.43	6.02	0.0019
Marinilabiliaceae	3.00	5.19	0.0060
Enterobacteriaceae	1.50	5.79	0.0001
Stappiaceae	0.77	4.04	0.0002
Leptotrichiaceae	0.99	3.22	0.0004
Pseudomonadaceae	1.77	2.15	0.1701
**Genus**			
Vibrio	23.18	7.25	0.0671
Photobacterium	23.40	4.89	0.0631
Pseudoalteromonas	6.54	7.15	0.9119
Aquimarina	1.34	8.37	0.2132
Paraburkholderia	3.96	4.93	0.8139
Carboxylicivirga	3.00	4.79	0.5504
Fusibacter	1.10	6.53	0.1728
Enterobacter	1.43	5.61	0.0116
Nautella	3.57	1.93	0.2330
Labrenzia	0.77	4.04	0.0954

Note: Relative abundances of each group are presented as mean value.

Table S4

The PERMANOVA showed the difference of intestinal microflora between DIV1-infected group and PBS group.

Group comparison	*F* value	*P* value	Significant
DIV1 vs PBS	1.8605	0.031	*

Dissimilar letters show significant difference ($P < 0.05$).

Table S5

Summary of RNA-seq data for *M. ensis* with and without DIV1.

Samples	Raw Reads Number	Clean Reads Number	Clean Bases (Gb)	Q20 (%)	Q30 (%)	Clean Reads Ratio (%)	GC (%)
DIV1-infected	93,281,594	93,073,404	13,895,625,533	96.93	91.86	99.60	45.87
Control	43,366,058	43,194,564	6,442,009,361	97.50	93.99	99.77	47.52

Table S6

Candidate genes involved in *M. ensis* immune response against DIV1.

Category or gene ID	Homologs function	Species	log_2FC
**MAPK signaling pathway - fly**			
Unigene0000434	Tyrosine-protein kinase SRK2-like	*Penaeus vannamei*	3.65
Unigene0010301	Ras-related protein ced-10-like	*Penaeus vannamei*	3.30
Unigene0019567	Dual oxidase	*Penaeus vannamei*	-2.91
Unigene0021886	Serine proteinase inhibitor	*Penaeus vannamei*	-2.96
Unigene0026988	Metabotropic glutamate receptor 3-like	*Penaeus vannamei*	-5.02
Unigene0033528	Tyrosine-protein kinase SRK3-like isoform X1	*Penaeus vannamei*	3.17
Unigene0033711	Protein spitz	*Penaeus vannamei*	-1.99
Unigene0033732	Transcription factor AP-1-like	*Penaeus vannamei*	3.16
Unigene0034054	Importin-7-like isoform X1	*Penaeus vannamei*	-1.53
**Wnt signaling pathway**			
Unigene0004417	Wnt16	*Penaeus vannamei*	3.20
Unigene0007294	Calcyclin-binding protein-like	*Penaeus vannamei*	-1.64
Unigene0010301	Ras-related protein ced-10-like	*Penaeus vannamei*	3.30
Unigene0015997	Protein chibby homolog 1-like	*Penaeus vannamei*	-1.22
Unigene0021448	Frizzled-5-like	*Penaeus vannamei*	4.35

Unigene0021484	Tyrosine-protein kinase RYK-like	*Penaeus vannamei*	-4.62
Unigene0028927	Frizzled-10-like	*Penaeus vannamei*	3.27
Unigene0033472	Frizzled-4-like	*Penaeus vannamei*	2.98
Unigene0033732	Transcription factor AP-1-like	*Penaeus vannamei*	3.16
**Peroxisome**			
Unigene0000404	Peroxiredoxin	*Penaeus monodon*	-2.96
Unigene0002522	Peroxisome biogenesis factor 1-like	*Penaeus vannamei*	-1.23
Unigene0024292	D-amino-acid oxidase-like	*Penaeus vannamei*	3.27
Unigene0033638	Peroxisomal membrane protein 2-like	*Penaeus vannamei*	-3.26
Unigene0033696	Peroxisome assembly factor 2-like	*Penaeus vannamei*	-1.51
Unigene0035960	Peroxisomal trans-2-enoyl-CoA reductase-like	*Penaeus vannamei*	-2.24
Unigene0036479	Peroxisomal sarcosine oxidase-like	*Penaeus vannamei*	3.64
**p53 signaling pathway**			
Unigene0016297	Cdc2	*Penaeus vannamei*	-3.15
Unigene0021511	Cyclin E	*Penaeus vannamei*	-1.54
Unigene0025560	Serine-protein kinase ATM-like	*Penaeus vannamei*	-2.08
Unigene0028364	Cyclin-dependent kinase 4-like	*Penaeus vannamei*	-1.39
Unigene0030693	Checkpoint kinase 1	*Penaeus monodon*	-4.35
Unigene0032219	Cyclin-dependent kinase 2-like	*Penaeus vannamei*	-3.10
Unigene0041670	Cyclin b	*Metapenaeus ensis*	-5.48
**C-type lectin receptor signaling pathway**			
Unigene0001950	C-type lectin 3	*Palaemon modestus*	4.69
Unigene0013250	Zinc finger protein 865-like	*Penaeus vannamei*	3.11
Unigene0024418	C-type lectin domain family 6 member A-like	*Penaeus vannamei*	3.99
Unigene0031532	Calcineurin subunit A	*Eriocheir sinensis*	-1.10
Unigene0033732	Transcription factor AP-1-like	*Penaeus vannamei*	3.16
Unigene0042667	NF-κB inhibitor cactus-like	*Penaeus vannamei*	2.87
**Toll-like receptor signaling pathway**			
Unigene0005987	Toll interacting protein	*Penaeus japonicus*	2.86
Unigene0010301	Ras-related protein ced-10-like	*Penaeus vannamei*	3.30
Unigene0032655	Rac1	*Cherax quadricarinatus*	-1.49
Unigene0033732	Transcription factor AP-1-like	*Penaeus vannamei*	3.16
Unigene0042667	NF-κB inhibitor cactus-like	*Penaeus vannamei*	2.87
**Toll and Imd signaling pathway**			
Unigene0019567	Dual oxidase	*Penaeus vannamei*	-2.91
Unigene0022277	Ankyrin-3-like isoform X4	*Penaeus vannamei*	2.60
Unigene0031614	Ubiquitin-conjugating enzyme E2	*Penaeus chinensis*	3.54

Unigene0033732	Transcription factor AP-1-like	*Penaeus vannamei*	3.16
Unigene0042667	NF-κB inhibitor cactus-like	*Penaeus vannamei*	2.87
**TNF signaling pathway**			
Unigene0013935	Caspase-1-like	*Penaeus vannamei*	2.96
Unigene0016565	Cyclic AMP response element-binding protein A-like	*Penaeus vannamei*	4.21
Unigene0029880	Caspase 2	*Penaeus chinensis*	-1.05
Unigene0033732	Transcription factor AP-1-like	*Penaeus vannamei*	3.16
Unigene0042667	NF-κB inhibitor cactus-like	*Penaeus vannamei*	2.87
**NOD-like receptor signaling pathway**			
Unigene0016933	STING-like protein	*Penaeus vannamei*	3.48
Unigene0023169	Protein SGT1 homolog	*Penaeus vannamei*	-1.97
Unigene0031122	Cathepsin B	*Penaeus japonicus*	-1.35
Unigene0031628	Heat shock protein 90	*Metapenaeus ensis*	-3.51
Unigene0033732	Transcription factor AP-1-like	*Penaeus vannamei*	3.16
Unigene0042667	NF-κB inhibitor cactus-like	*Penaeus vannamei*	2.87
**Apoptosis**			
Unigene0011013	Caspase 4	*Penaeus vannamei*	2.90
Unigene0013935	Caspase-1-like	*Penaeus vannamei*	2.96
Unigene0024777	Cathepsin L precursor	*Metapenaeus ensis*	-9.59
Unigene0025560	Serine-protein kinase ATM-like	*Penaeus vannamei*	-2.08
Unigene0029880	Caspase 2	*Penaeus chinensis*	-1.05
Unigene0031122	Cathepsin B	*Penaeus japonicus*	-1.35
Unigene0033367	Tubulin alpha-1A chain-like	*Penaeus vannamei*	4.13
Unigene0033732	Transcription factor AP-1-like	*Penaeus vannamei*	3.16
Unigene0042667	NF-κB inhibitor cactus-like	*Penaeus vannamei*	2.87
**Lysosome**			
Unigene0005380	Sphingomyelin phosphodiesterase-like	*Penaeus vannamei*	-5.61
Unigene0006099	Dipeptidyl peptidase 1-like	*Penaeus vannamei*	-3.20
Unigene0008979	Glucosylceramidase-like	*Penaeus vannamei*	-3.47
Unigene0010876	Lysosome membrane protein 2-like	*Penaeus vannamei*	3.91
Unigene0013491	Battenin-like isoform X1	*Penaeus vannamei*	-1.60
Unigene0024777	Cathepsin L precursor	*Metapenaeus ensis*	-9.59
Unigene0025407	Protein patched homolog 2-like	*Penaeus vannamei*	3.33
Unigene0031122	Cathepsin B	*Penaeus japonicus*	-1.35
Unigene0034391	Sucrase-isomaltase, intestinal-like	*Penaeus vannamei*	3.31
**PI3K-Akt signaling pathway**			
Unigene0000587	Laminin subunit alpha-like	*Penaeus vannamei*	-3.65
Unigene0000785	Integrin beta-PS-like	*Penaeus vannamei*	2.77

Unigene0001399	Endoplasmin	*Penaeus monodon*	-2.95
Unigene0001903	Integrin alpha-IIb-like	*Penaeus vannamei*	4.20
Unigene0006152	Integrin alpha 8	*Penaeus chinensis*	2.93
Unigene0010301	Ras-related protein ced-10-like	*Penaeus vannamei*	3.30
Unigene0011123	Thrombospondin	*Penaeus vannamei*	-6.18
Unigene0011702	Insulin-like receptor	*Macrobrachium rosenbergii*	3.05
Unigene0016565	Cyclic AMP response element-binding protein A-like	*Penaeus vannamei*	4.21
Unigene0018486	Integrin beta-PS-like	*Penaeus vannamei*	3.30
Unigene0021511	Cyclin E	*Penaeus vannamei*	-1.54
Unigene0022776	Glucose-6-phosphatase-like	*Penaeus vannamei*	4.10
Unigene0026169	Serine/threonine protein phosphatase Ppa2	*Penaeus vannamei*	3.99
Unigene0027549	Serine/threonine-protein kinase Sgk3	*Penaeus vannamei*	3.62
Unigene0028364	Cyclin-dependent kinase 4-like	*Penaeus vannamei*	-1.39
Unigene0031628	Heat shock protein 90	*Metapenaeus ensis*	-3.51
Unigene0032219	Cyclin-dependent kinase 2-like	*Penaeus vannamei*	-3.10
**Phagosome**			
Unigene0000189	Tubulin beta-1 chain	*Penaeus vannamei*	-3.05
Unigene0000785	Integrin beta-PS-like	*Penaeus vannamei*	2.77
Unigene0010301	Ras-related protein ced-10-like	*Penaeus vannamei*	3.30
Unigene0011123	Thrombospondin	*Penaeus vannamei*	-6.18
Unigene0016686	Nitric oxide synthase	*Penaeus vannamei*	2.80
Unigene0018486	Integrin beta-PS-like	*Penaeus vannamei*	3.30
Unigene0022418	Tubulin beta-2 chain	*Penaeus vannamei*	-3.69
Unigene0024777	Cathepsin L precursor	*Metapenaeus ensis*	-9.59
Unigene0033445	Class B scavenger receptor	*Eriocheir sinensis*	-4.39
Unigene0034115	Calreticulin	*Penaeus monodon*	-3.37
Unigene0038962	Tubulin	*Penaeus vannamei*	2.77
Unigene0038963	Tubulin beta chain-like	*Penaeus vannamei*	3.36

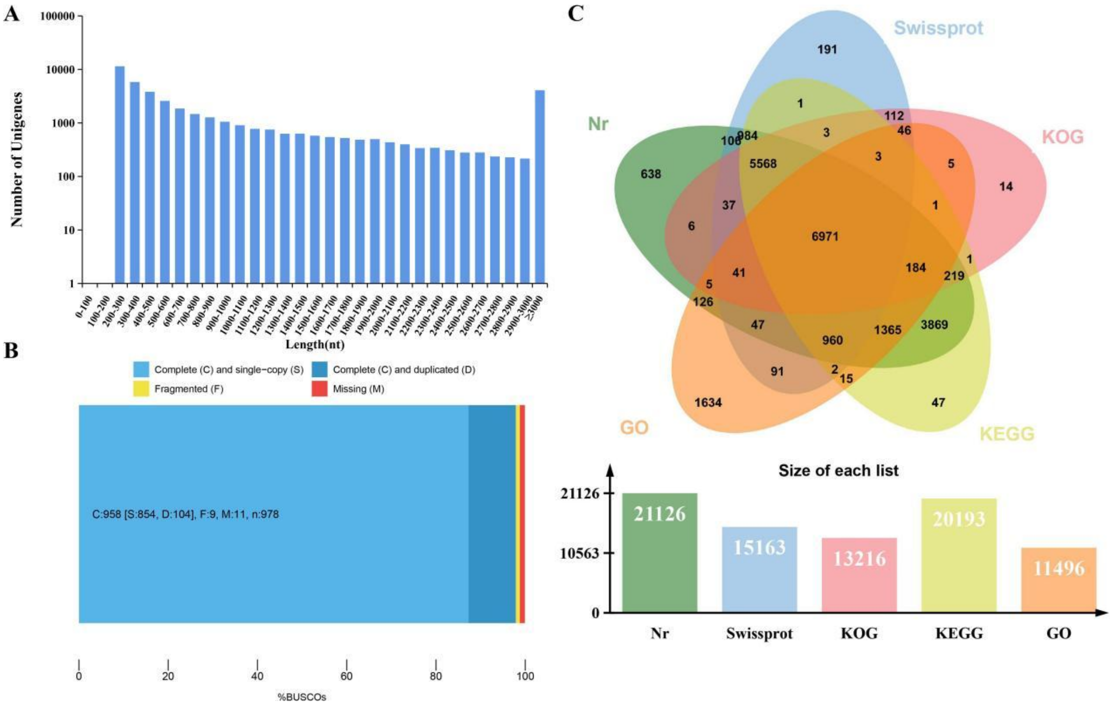

Fig. S1. Length distribution, assembly quality, and annotation analysis of unigenes in *M. ensis* gut transcriptome. (A) The x-axis shows the interval length of unigenes, and the y-axis shows the number of unigenes. (B) Analysis of BUSCOs. (C) The Venn diagram shows statistics on the successful annotation of unigenes in five databases.

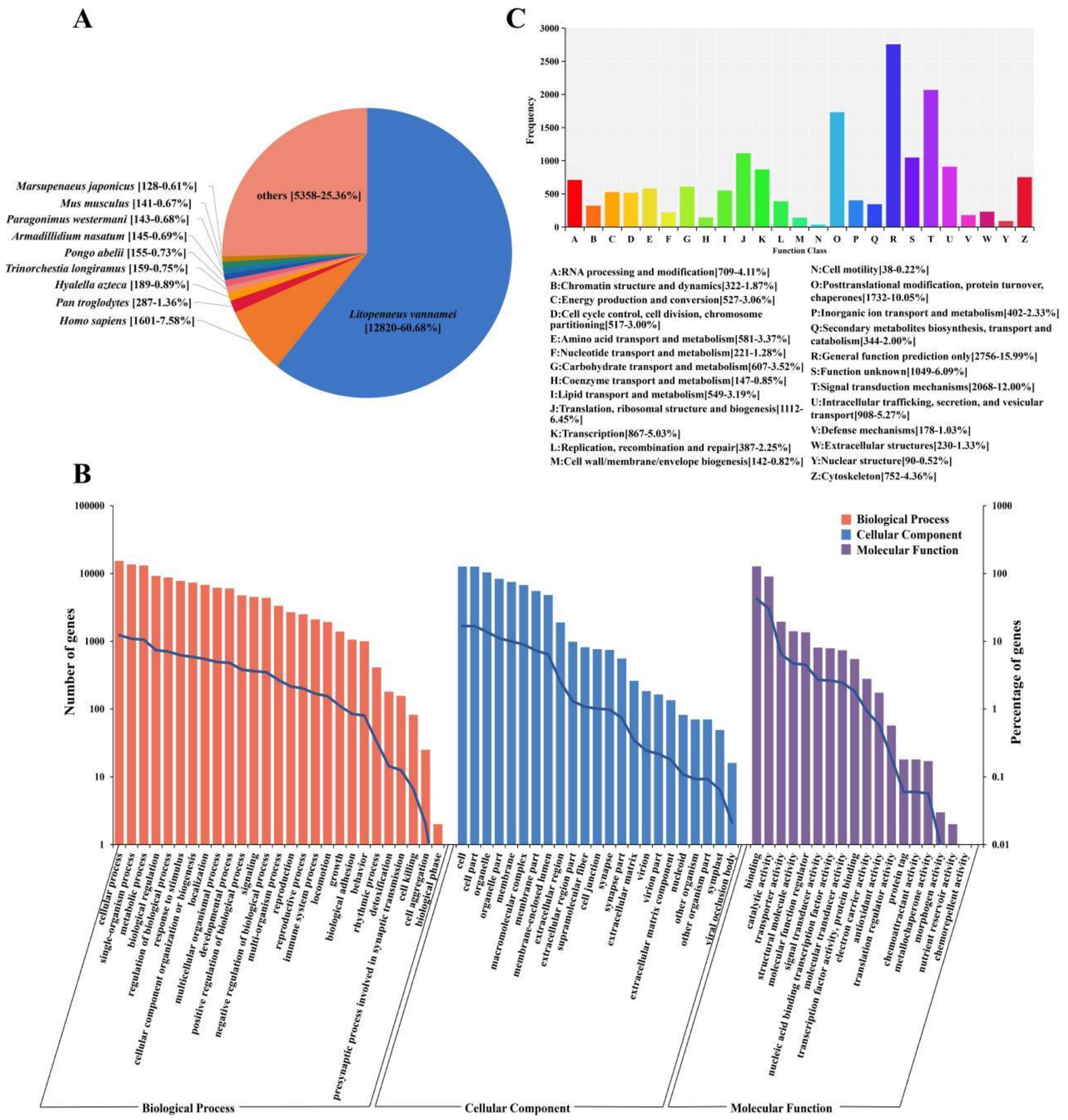

Fig. S2. Sequence analysis and functional enrichment of unigenes from the *M. ensis* intestine. (A) Species distribution of transcriptome unigenes annotated in Nr database. (B) Gene ontology (GO) annotation of *M. ensis* intestine. The x-axis indicates 3 GO functional classifications with 68 subcategories. The y-axis on the left indicates the number of annotated genes, and y-axis on the right indicates the percentage of annotated genes. (C) KOG functional annotation in the transcriptome. The x-axis stands for the functional categories, and y-axis indicates the frequency.

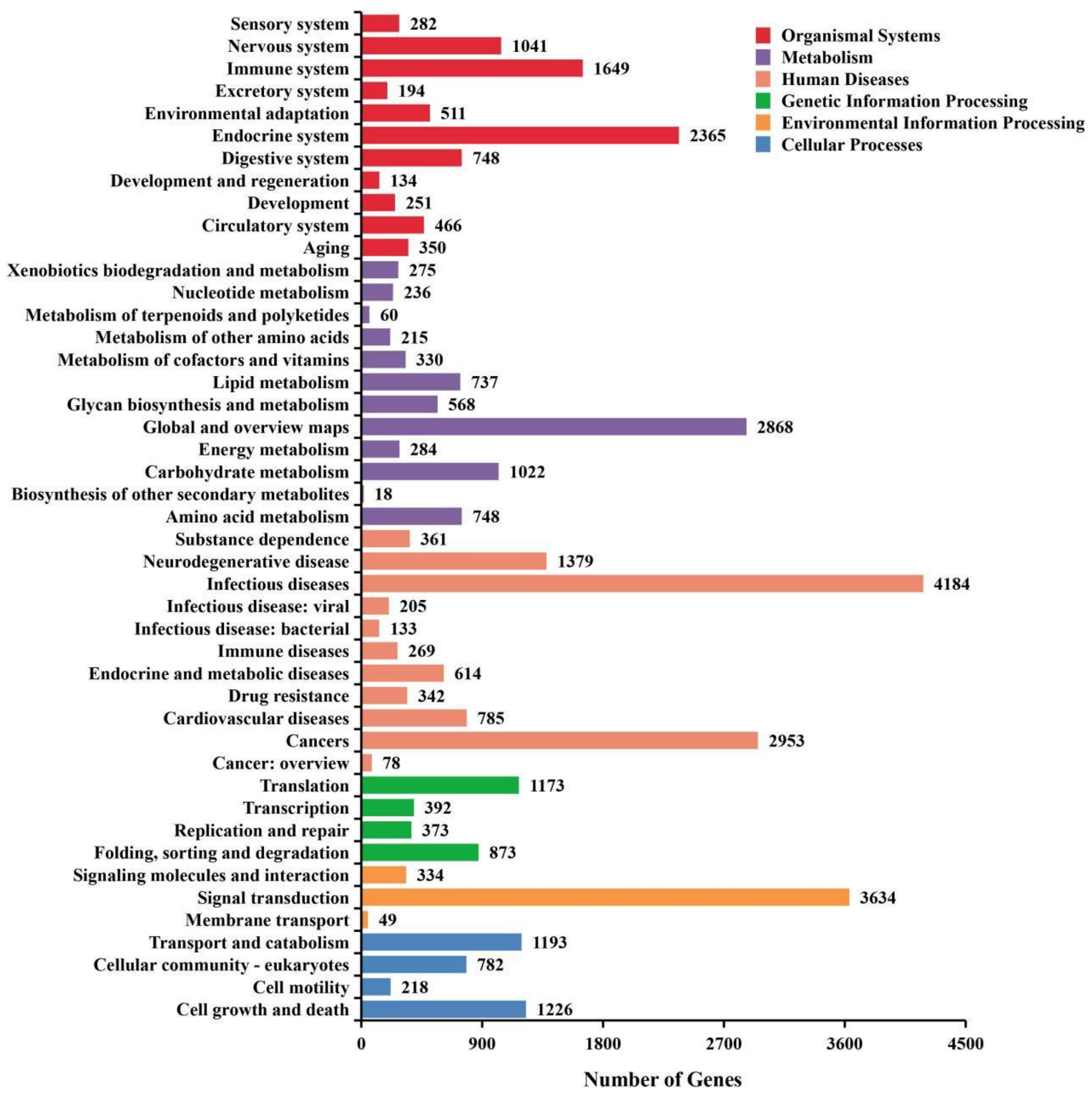

Fig. S3. The KEGG biological pathway classification histograms for annotated unigenes identified in the intestine of *M. ensis*. Each bar represents the number of unigenes classified into different biological processes. The x-axis shows the number of genes, while the y-axis shows the pathways from the KEGG classification.
